# A Specific CD44lo CD25lo Subpopulation of Regulatory T Cells Inhibits Anti-Leukemic Immune Response and Promotes the Progression in a Mouse Model of Chronic Lymphocytic Leukemia

**DOI:** 10.3389/fimmu.2022.781364

**Published:** 2022-02-28

**Authors:** Agnieszka Goral, Malgorzata Firczuk, Klaudyna Fidyt, Marta Sledz, Francesca Simoncello, Karolina Siudakowska, Giulia Pagano, Etienne Moussay, Jérôme Paggetti, Patrycja Nowakowska, Stefania Gobessi, Joanna Barankiewicz, Aleksander Salomon-Perzynski, Federica Benvenuti, Dimitar G. Efremov, Przemyslaw Juszczynski, Ewa Lech-Maranda, Angelika Muchowicz

**Affiliations:** ^1^ Department of Immunology, Medical University of Warsaw, Warsaw, Poland; ^2^ Cellular Immunology, International Centre for Genetic Engineering and Biotechnology, Trieste, Italy; ^3^ Tumor-Stroma Interactions, Department of Cancer Research, Luxembourg Institute of Health, Luxembourg, Luxembourg; ^4^ Molecular Hematology, International Centre for Genetic Engineering and Biotechnology, Trieste, Italy; ^5^ Department of Experimental Hematology, Institute of Hematology and Transfusion Medicine, Warsaw, Poland

**Keywords:** Tregs, CLL, Eµ-TCL1, MALT1, TCR repertoire, anti-leukemic immune response

## Abstract

Regulatory T cells (Tregs) are capable of inhibiting the proliferation, activation and function of T cells and play an important role in impeding the immune response to cancer. In chronic lymphocytic leukemia (CLL) a dysfunctional immune response and elevated percentage of effector-like phenotype Tregs have been described. In this study, using the Eµ-TCL1 mouse model of CLL, we evaluated the changes in the Tregs phenotype and their expansion at different stages of leukemia progression. Importantly, we show that Tregs depletion in DEREG mice triggered the expansion of new anti-leukemic cytotoxic T cell clones leading to leukemia eradication. In TCL1 leukemia-bearing mice we identified and characterized a specific Tregs subpopulation, the phenotype of which suggests its role in the formation of an immunosuppressive microenvironment, supportive for leukemia survival and proliferation. This observation was also confirmed by the gene expression profile analysis of these TCL1-specific Tregs. The obtained data on Tregs are consistent with those described so far, however, above all show that the changes in the Tregs phenotype described in CLL result from the formation of a specific, described in this study Tregs subpopulation. In addition, functional tests revealed the ability of Tregs to inhibit T cells that recognize model antigens expressed by leukemic cells. Moreover, inhibition of Tregs with a MALT1 inhibitor provided a therapeutic benefit, both as monotherapy and also when combined with an immune checkpoint inhibitor. Altogether, activation of Tregs appears to be crucial for CLL progression.

## Introduction

Despite the extensive research and the development of new treatment modalities, the number of chronic lymphocytic leukemia (CLL) cases with clinical resistance to therapy is constantly rising (1). The newest achievement in immunotherapy – chimeric antigen receptor T cells (CAR-T cells) – are less effective in CLL as compared to other B cell malignancies, including B cell acute lymphoblastic leukemia or diffuse large B cell lymphoma (2–4). Similarly, immune checkpoint inhibitors have a limited efficacy in relapsed/refractory CLL (3). In preclinical studies, antibodies against lymphocyte activation gene 3 (LAG-3), programmed cell death protein 1 (PD-1) or programmed death-ligand 1 (PD-L1) are only effective when administrated in the initial stage of leukemia development (5–7). Importantly, the immune system dysfunctions observed in CLL patients, suggest that CLL cells modulate the microenvironment to their own benefit (8–10). The exhausted phenotype of T cells that display high expression of PD-1, LAG-3, or T cell immunoglobulin domain and mucin domain (TIM-3) is a hallmark of CLL (11, 12). In order to improve the therapeutic strategies for CLL, it is crucial to understand the mechanisms that shape the leukemia microenvironment.

Naturally occurring, thymic, Forkhead box protein P3 (FoxP3)^+^, CD4^+^ regulatory T cells (Tregs), are sensitive to activation by self-antigens and tumor neoantigens, and are main players of the neoplastic microenvironment (13). Tregs can affect T cells in all stages of immune response development: priming, proliferation, and T cell effector functions (14). Increased frequency of Tregs correlates with poor prognosis of CLL patients (15). The expression patterns of Tregs-associated markers (CD25, LAG-3, killer cell lectin like receptor G1, CD69, Eomesodermin - EOMES) that determines their suppressive functions was recently presented in both CLL patients and leukemia-bearing mice (5, 16, 17). Nevertheless, the function of Tregs in CLL has not been elucidated and the approaches for Tregs elimination have shown to be insufficient. For instance, the administration of anti-CD25 antibodies or phosphoinositide 3-kinase δ (PI3Kδ) inhibitors affected not only Tregs but also abrogated the activation and function of CD8^+^ lymphocytes (18).

In order to evaluate the role of Tregs in the development and shaping of immunosuppressive microenvironment of CLL, in this work we used Eµ-TCL1 transgenic mice model (19, 20). We characterized a novel, TCL1-derived Tregs subpopulation and assessed Tregs suppressive activity in functional tests. Furthermore, TCR sequencing allowed us to better understand the influence of leukemia on Tregs and CD8^+^ T lymphocytes activation and clonality. Finally, we used the inhibitor of mucosa-associated lymphoid tissue lymphoma translocation protein 1 (MALT1) to block the activation of Tregs. MALT1 protease is a component of CARMA1-BCL10-MALT1 (CBM) complex which was shown to be crucial for Tregs activity (21). The results obtained in this study provide the evidence that Tregs are essential for leukemia progression in immunocompetent mice and can be efficiently targeted to block CLL progression.

## Materials And Methods

### Reagents

MI-2 (Malt1 inhibitor, Selleckchem.com) was dissolved in DMSO (Sigma-Aldrich, St Louis, MA, USA), aliquoted and stored at -20°C. Albumin from chicken egg white (OVA, Sigma-Aldrich, St Louis, MA, USA) and Poly (I:C) (HMW) (*In vivo*Gen, San Diego, CA, USA) were aliquoted and stored at -20°C. Diphtheria Toxin (DT) from *Corynebacterium diphtheriae* (Sigma-Aldrich, St Louis, MA, USA) was aliquoted and stored at -80°C. Anti-mouse PD-L1 antibody InVivoPlus (B7-H1) (BioXcell, Lebanon, NH, USA) and InVivoPlus rat IgG2b isotype control, (BioXcell, Lebanon, NH, USA) were stored at 4°C.

### Animals Studies

All *in vivo* studies were performed in accordance with the EU Directive 2010/63/EU and the Polish legislation for animal experiments of the Polish Ministry of Science and Higher Education (February 26, 2015) and approved by the Local Ethics Committee for the Animal Experimentation in Warsaw. The *in vivo* experiments were carried out in Animal Facility of the Medical University of Warsaw.

For the study, 6-12 weeks old female or male (never mixed in one experiment) mice were used. Mouse strains include: C57BL6/J (wild-type, immunocompetent mice) (Medical University of Bialystok or Mossakowski Medical Research Centre), B6.Cg-Foxp3tm2(EGFP)Tch/J (B6 Foxp3^EGFP^, Tregs express GFP) (University of Warsaw), C57BL/6-Tg(Foxp3-DTR/EGFP)23.2Spar/Mmjax (DEREG, Tregs express GFP and receptor for diphtheria toxin) (The International Centre for Genetic Engineering and Biotechnology, Trieste, Italy) B6(Cg)-Rag2tm1.1Cgn/J (RAG2-KO, immunodeficient mice) and C57BL/6-Tg(TcraTcrb)1100Mjb/J (OT-1) (Medical University of Warsaw). Splenocytes or leukemic CD5^+^CD19^+^ TCL1 cells (5×10^6^ – 1×10^7^) isolated from spleens of female Eμ-TCL1 transgenic mice (The International Centre for Genetic Engineering and Biotechnology, Trieste, Italy) were adoptively transferred *via* tail vein injection. In described experiments we used cells isolated from two different Eµ-TCL1 transgenic mice, either TCL1-1159 or TCL1-1013. These cells were propagated in mice maximally twice, with the exception of genetically modified TCL1 cells expressing OVA (due to the procedure of generating modified cells, they required additional propagation in RAG2-KO mice).

### *In Vivo* Treatments

Eµ-TCL1 mice model of CLL was used in this study. To monitor leukemia development and progression, the percentage of leukemic TCL1 cells (CD5^+^CD19^+^) among white blood cells (WBC) in the peripheral blood (PB) collected from cheek vein was assessed by flow cytometry. The consistency in the assessment of leukemia was ensured and blinding practice was not applicable. Mice with detected leukemia were randomly selected and further used in the experiments. The sample size was calculated with power analysis test (22).

DEREG mice were treated with DT (50 µg/kg) administered intraperitoneally (i.p.) every four days. MI-2 was administered i.p. daily, at dose 20 mg/kg and the control mice were injected with the DMSO as a solvent. Anti-PD-L1 antibody or the appropriate isotype control were administered i.p. every second day at a dose 200 μg/mouse. The schemes of the treatments are presented in details in the appropriate figures.

### Cell Isolation

In order to prepare a single cell suspension, spleens (SPL) or lymph nodes (LNs) were cut in small pieces and passed through a 150 μm cell strainer. To remove red blood cells the isolated splenocytes were lysed with ACK Lysing Buffer (Thermofisher Scientific, Waltham, MA, USA) according to the manufacturer’s instructions. CD19^+^, CD4^+^ and CD8^+^ cell subpopulations were isolated by immunomagnetic negative selection using EasySep™ Mouse B Cell Isolation Kit, EasySep™ Mouse CD4^+^ T cell Isolation Kit and EasySep™ Mouse CD8^+^ Cell Isolation Kit (STEMCELL Technologies, Vancouver, Canada), respectively, according to the manufacturer’s protocols. The efficacy of the isolation was over 90%.

### CD8^+^ Cells Proliferation Assay

CD8^+^ cells isolated from spleens were incubated with CellTrace ™ Violet Cell Proliferation kit (CT) (Invitrogen/Thermo Fisher Scientific, Waltham, MA, USA) for 20 min at 37°C, washed with cell culture medium and seeded onto 96-well U-bottom plates coated with anti-CD3 antibody (eBioscience, San Diego, CA, USA) together with sorted Tregs-GFP (either all GFP^+^ or GFP^+^ CD69^high^ CD44^-/low^) in various ratios (1:0.125, 1:0.25, 1:0.5, 1:1 and 1:2). For stimulation, anti-CD28 (eBioscience, San Diego, CA, USA) antibody was added to the culture medium. The proliferation of CD8^+^ cells was evaluated upon 72h using BD FACSCanto™ II Flow Cytometer and BD FACSDiva Software (v8.0.1) (BD Biosciences, La Jolla, CA, USA).

### TCL1 OVA-Expressing Cells

The sequence encoding ovalbumin (Addgene, cat. number 25097) was inserted into mammalian expression vector pCDH-EF1-MCS-T2A-copGFP (System Biosciences). The pCDH-EF1-OVA-GFP and a packaging (psPAX2) and an envelope (pMD2.G) plasmids (gifts from prof. Didier Trono, École Polytechnique Fédérale de Lausanne, Switzerland) were introduced into HEK-293T cells using Polyethylenimine (Polysciences). Then freshly isolated TCL1 cells (CD5^+^CD19^+^) from mouse spleens were seeded into 24-well plates with M2-10B4 murine stroma cells. Next, medium containing lentiviral particles was added into TCL1 and M2-10B4 cells co-culture. Then TCL1 cells were washed and inoculated into RAG2-KO mice for leukemic cells propagation. Finally, OVA^+^ GFP^+^ cells were sorted and used for further experiments. In all performed experiments at least 60% of injected leukemic cells exerted OVA^+^ GFP^+^ phenotype as evaluated by flow cytometry.

### *In Vivo* Functional Assays

Two weeks following TCL1 cells adoptive transfer, leukemia-bearing DEREG transgenic mice were treated with DT and on the following day, injected with CT-positive CD8^+^ T cells isolated from spleens and lymph nodes of OT-1 mice. 4-5 hours later, the mice were i.v. inoculated with OVA protein (50 µg). The proliferation of CD8^+^ OT-1 cells isolated from spleens was assessed upon 3 days using flow cytometry. In the second approach, DEREG mice were injected with genetically modified TCL1 leukemic cells expressing OVA-GFP (TCL1-OVA). Three days later, the mice were treated with DT and on the following day, injected with CT-positive CD8^+^ OT-1 T cells. The proliferation of CD8^+^ OT-1 cells was evaluated following 3 or 4 days using flow cytometry. The schemes of described experiments are presented in detail on appropriate figures.

### Flow Cytometry

The isolated cells were stained with Zombie NIR™ Fixable Viability kit or Zombie Violet™ Fixable Viability Kit (BioLegend, San Diego, CA, USA) for 20 min at room temperature (RT) and washed with PBS. Next, the cells were incubated with Purified Rat Anti-Mouse CD16/CD32 (Mouse BD Fc Block™; clone 2.4G2, BD Biosciences, La Jolla, CA, USA) for 15 min at RT and stained for surface markers with proper fluorochrome-conjugated antibodies (all antibodies used in this study are listed in **Supplementary Table 1**) for 20-30 min at RT. After final washing with PBS, the cells were analysed using BD FACS Canto™ II Flow Cytometer and BD FACS Diva Software (v8.0.1)(BD Biosciences, La Jolla, CA, USA). For further analyses, including t-SNE (with markers: CD44, CD25, LAG-3, CD69), FlowJo Software (v. 10.6.1) (FlowJo LLC, Ashland, OR, USA) was used.

### Cell Sorting

In order to sort Tregs (CD4^+^, GFP^+^) from spleens of B6 Foxp3^EGFP^ or DEREG mice, CD4^+^ cell subpopulation was enriched prior to sorting. To this end, isolated splenocytes were subjected to immunomagnetic positive selection for CD19^+^ using EasySep™ Mouse CD19 Positive Selection Kit II (STEMCELL Technologies) and then the negative fraction was subsequently subjected to negative selection using EasySep™ Mouse CD4^+^ T cell Isolation Kit (STEMCELL Technologies). When needed, CD4^+^ cells were additionally stained with anti-CD69-PE and anti-CD44-PE-Cy7 monoclonal antibodies as described above. To sort CD8^+^ cells, the fraction of splenocytes devoid of CD19^+^ cells was stained with anti-CD8a-PerCP-Cy5.5 monoclonal antibody. Then the cells were sorted using BD FACS Aria™ III Cell Sorter (BD Biosciences).

### DNA Isolation and Analysis of TCRβ Repertoire

Tregs A (GFP^+^, CD69^high^, CD44^-/low^, gated as presented in **Figure 5** CD69/CD44 right panel) and CD8^+^ cells were sorted as described above. Then the genomic DNA was isolated from the sorted cells using DNA Micro Kit (QIAGEN, Hilden, Germany) according to the manufacturer’s instructions. The concentration and purity of extracted DNA was assessed using NanoDrop 2000 Spectrophotometer (Thermo Fisher Scientific). Immunosequencing of the CDR3 regions of TCRβ chains was performed with immunoSEQ^®^ Assay and analysed by immunoSEQ^®^ Analyzer (Adaptive Biotechnologies, Seatlle, WA, USA).

### RNA Sequencing

When percentage of leukemic cells in mouse blood reached at least 20% of all PBMC, the GFP^+^ Tregs: A (GFP^+^, CD69^high^, CD44^-/low^) and B (GFP^+^ excluding fraction A) were sorted from TCL1 leukemia-injected DEREG mice. Additionally, GFP^+^ Tregs were also sorted from control DEREG mice. The mRNA was isolated from 4.5 × 10^5^ cells with the RNeasy Micro Kit (Qiagen, Hilden, Germany). Libraries were prepared with the QuantSeq 3’ mRNA-Seq Library Prep Kit FWD for Illumina (Lexogen), according to manufacturer’s instructions, with the addition of UMI. Barcoded samples were pooled, diluted, loaded onto a NextSeq 500/550 Mid Output flowcell (130M reads, Illumina) and single-end 150bp sequencing was performed on a NextSeq 550 (Illumina).

After initial QCs using FastQC (https://www.bioinformatics.babraham.ac.uk/projects/fastqc/) and FastQ Screen (https://www.bioinformatics.babraham.ac.uk/projects/fastq_screen/), fastq files were processed using a local Snakemake workflow including the following main steps. First, raw reads were trimmed from their UMI index, poly A and adapter sequences using a combination of dedicated scripts and cutadapt (v2.10). Next, filtered reads were submitted for mapping (STAR v2.5.3a) on the Mouse Reference genome (GRCm38). Collapsing of reads originating from the same fragment was achieved with umi_tools (v 1.0.0) and counting was performed with featureCounts (subread v2.0.0).

Counts were filtered and transformed with edgeR (cpm > 5 and presence in at least 3 samples). For data visualization, heatmaps, sample distance matrix, and volcano plots were drawn with EdgeR, heatmap, and EnhancedVolcano R packages. For differential expression of genes across samples (DEGs), FDR < 0.05 and log2 fold change cut-off of 1 were imposed. For clustering, DEGs were selected as important for immune functions in Tregs. Gene expression values were z-scored and subjected to correlation-based clustering with complete linkage. Raw and processed data were deposited at the NCBI GEO database (GSE179121). The following secure token has been created to allow review of record GSE179121 while it remains in private status: qlchuysazpinjed. To better understand the nature of Tregs A and Tregs B, we re-analyzed the public dataset GSE72494 describing the transcriptome of naive, activated, and effector Treg (23) and performed a Gene Set Enrichment Analysis (GSEA, Hallmark and curated gene sets) with the stand-alone software (GSEA v4.2.1, Broad Institute, Boston, MA). Normalized enrichment scores (NES) and p-values < 0.05 were taken into consideration.

### Statistical Analysis

GraphPad Prism 6 Software (GraphPad Software Inc., San Diego, CA, USA) was used for data analysis. The statistical significance was calculated by Mann-Whitney U test. The mice survival rate was analyzed by log-rank survival test. For gene expression data (RNA sequencing), one-way ANOVA with multiple comparisons was calculated for single genes and histograms were drawn with GraphPad Prism 9 Software.

Additional experimental procedures are described in details in the **Supplementary Material**.

## Results

### Depletion of Tregs in Mice With Adoptively Transferred TCL1 Leukemia Results in the Expansion of Functional CD8^+^ Cells and Leukemia Clearance

**Figure 1:**
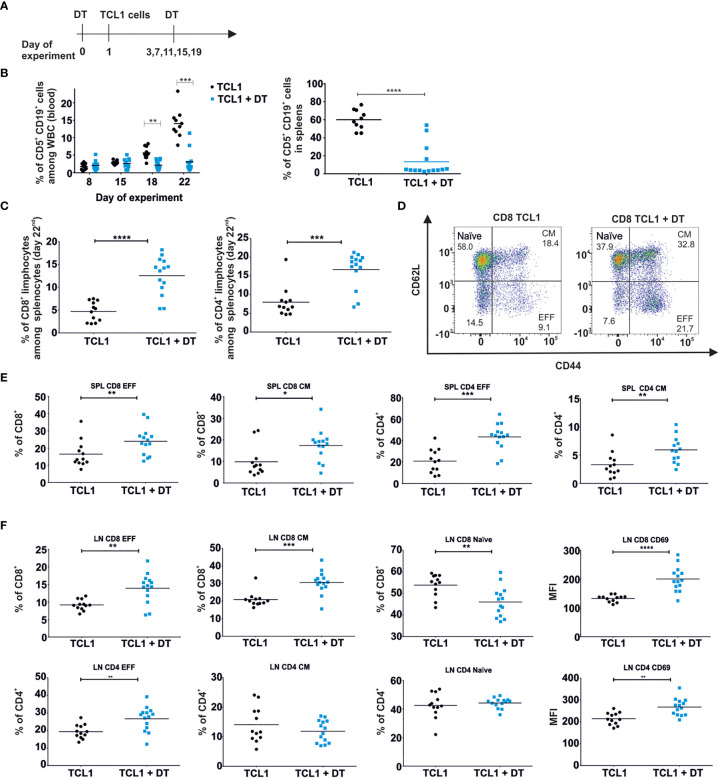
Depletion of Tregs diminishes the progression of leukemia in DEREG mice and affects the relative frequency of conventional T cell subpopulations. **(A)** The graph presenting a scheme of the experiment. Tregs were depleted with DT and on the next day mice were injected with TCL1 CD19^+^ leukemic cells. The depletion of Tregs (DT administration) was repeated every 4 days. **(B)** The percentage of leukemic cells (CD5^+^CD19^+^) among all white blood cells (WBC) assessed by flow cytometry in blood (at indicated time points of the experiment, left) and spleens (day 22^nd^ of experiment, right) of untreated (TCL1) and DT-treated (TCL1+DT) TCL1 leukemia-bearing mice. The graphs represent mean results from two independent experiments. Each dot represents an individual sample (mouse), n = 10-12, Mann-Whitney U test *p ≤ 0.05. **(C)** The Percentage of CD8^+^ (left) and CD4^+^ (right) T cells in spleens of untreated and DT-treated TCL1 leukemia-bearing mice. The graphs present mean results from two independent experiments. Each dot represents an individual sample (mouse), n = 12-14, Mann-Whitney U test ***p ≤ 0.001, ****p < 0.0001. **(D–F)** The percentage of CD4^+^ and CD8^+^ T cells with phenotype of naïve, effector (EFF) and central memory (CM) subpopulations according to the expression of CD44 and CD62L surface markers. Representative dot plots with a gating strategy **(D)** and the graphs present the results from spleens (SPL) **(E)** and lymph nodes (LN) **(F)** of untreated and DT-treated TCL1 leukemia-bearing mice. In **(F)** the graphs presenting the expression of CD69 surface marker on CD4^+^ and CD8^+^ T cells in LN are also shown. The data from two independent experiments are showing mean values. Each dot represents an individual sample (mouse), n = 12-14, Mann-Whitney U test *p ≤ 0.05, **p ≤ 0.01, ***p ≤ 0.001, ****p < 0.0001.

To evaluate the significance of Tregs for CLL progression we used DEREG transgenic mice with depletion of FoxP3^+^ CD4^+^ Tregs by treatment with diphtheria toxin (DT) (**Figure 1** and **Supplementary Figure 1A, B**). DEREG mice were treated with DT one day prior to adoptive transfer of malignant (CD5^+^CD19^+^) B cells, isolated from an Eµ-TCL1 transgenic mouse. Effective depletion of Tregs was observed in spleens and peripheral blood of DEREG mice and was maintained by additional DT injections every four days (**Figure 1A** and **Supplementary Figure 1A**). As monitored in peripheral blood twice a week, injection of DT did not affect the progression of leukemia during the first fifteen days of experiments. However, starting from day 18^th^ after TCL1 leukemia inoculation, we detected a significant decrease in the percentage of leukemic cells (CD5^+^CD19^+^), in the peripheral blood, of DT-treated mice as compared to untreated TCL1 leukemia-bearing animals (**Figure 1B**, left panel). In line with these results, we observed a significant reduction of previously established leukemia in the spleens of Tregs-depleted mice (**Figure 1B**, right panel). The same observations were made when DEREG mice were injected with TCL1 leukemia isolated from another transgenic mouse (**Supplementary Figure 1B**). The decrease in the percentage of leukemic cells in spleens of DT-treated mice was accompanied by the extensive increase of the percentage in both CD4^+^ and CD8^+^ T lymphocytes (**Figure 1C**). These observations prompted us to investigate the putative changes in the phenotype of splenic CD4^+^ and CD8^+^ T cells mediated by Tregs-depletion. We observed the enrichment of effector (EFF; CD44^+^CD62L^-^) and central memory (CM; CD44^+^CD62L^+^) cells in both CD4^+^ and CD8^+^ T cell subpopulations in mice deprived of Tregs (**Figures 1D, E**). A significant increase in the percentage of effector (CD4^+^ and CD8^+^) and central memory (CD8^+^) T cells was also observed in lymph nodes (axillary, brachial, inguinal) of DT-treated mice (**Figure 1F**). Depletion of Tregs resulted in the elevation of CD69 on both, CD4^+^ and CD8^+^ T cells in the lymph nodes, and reduced the percentage of naïve cells, suggesting the activation of a systemic immune response. Nevertheless, the depletion of Tregs, performed at an advanced stage of the disease (first dose of DT was administered when 30% of malignant B cells were detected among all white blood cells in peripheral blood) did not affect the progression of leukemia (**Supplementary Figure 1C**). Tregs depletion at an advanced stage of leukemia progression increased the percentage of effector and IFN-γ-positive CD4^+^ and CD8^+^ T lymphocytes, significantly elevated IFN-γ concentration and reduced the concentration of IL-10 in the sera (**Supplementary Figure 1D, E**). Importantly, three weeks of DT injections of control (without leukemia) DEREG mice lead to a minor activation of T cells, mostly CD4^+^ T cell subpopulation (**Supplementary Figure 1F**, upper panel). However, no changes were observed in the level of CD69 in lymphatic T cells upon DT-treatment (**Supplementary Figure 1F**, lower panel). The activation of CD4^+^ T cells may be the result of anti-DT immune response as it was described before (24).

**Figure 2:**
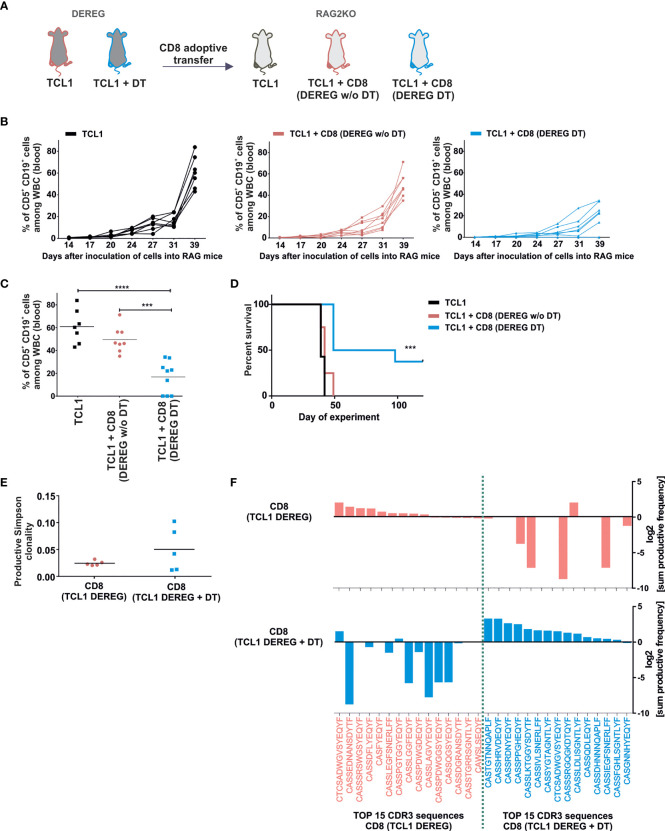
Depletion of Tregs in TCL1 leukemia-bearing DEREG mice results in the expansion of CD8+ lymphocytes capable of eradicating leukemic cells. **(A)** The graph presenting a scheme of the experiment. DEREG mice were treated according to scheme from Fig1A. Then 5 x 10^6^ of splenic CD8^+^ T cells isolated with magnetic beads from untreated (DEREG w/o DT) or DT-treated (DEREG DT) leukemic DEREG mice were injected to RAG2-KO mice following the injection of TCL1 (CD5^+^CD19^+^) cells. **(B, C)** The percentage of leukemic cells (CD5^+^CD19^+^) assessed at indicated time points in the peripheral blood of RAG2-KO mice: TCL1 leukemia-injected mice (black lines), TCL1- and CD8^+^-injected mice (CD8^+^ isolated from TCL1 leukemia bearing-DEREG w/o DT, pink lines), and TCL1- and CD8^+^-injected mice (CD8^+^ isolated from leukemia-bearing DEREG treated with DT, blue lines). Each line represents an individual sample (mouse), **(B)** and on day 39^th^ **(C)**, each dot represents an individual sample (mouse). The graphs represent mean results from two independent experiments, n = 7-9, Mann-Whitney U test ***p ≤ 0.001, ****p < 0.0001. **(D)** The survival plot summarizing the results from two independent experiments, n = 7-9, log-rank survival test ***p ≤ 0.001. **(E)** The productive Simpson clonality of CD8^+^ lymphocytes sorted from untreated or DT-treated mice analyzed in immunoSEQ Analyzer (from Adaptive Biotechnologies), n = 5. **(F)** The top 15 amino acid sequences of CDR3 TCRβ with the highest sum frequency (total amount of clones with a given sequence in all tested mice), of CD8^+^ lymphocytes sorted from untreated (pink) or DT-treated TCL1 leukemia-bearing DEREG mice (blue). The Graphs present log2 transformation of % sum frequency of a given sequence in untreated (pink, upper graph) and DT- treated TCL1- injected DEREG mice (blue, lower graph), n = 5.

To understand more deeply the anti-leukemia immune response induced by Tregs depletion, we investigated the impact of CD8^+^ T lymphocytes derived from the mice after DT injections on leukemia progression. We limited these experiments to the subset of CD8^+^ T cells as it was shown that these cells play a superior role in anti-leukemia immune response over CD4^+^ T lymphocytes (25). Importantly, an effective Tregs depletion in DEREG mice is transient. At day 22 after TCL1 injection we observed that the Tregs population was restored in murine blood despite continuous injections of DT (**Supplementary Figure 1A**, right panel), as was also reported by others (26). Thus, to examine the impact of the CD8^+^ lymphocytes on leukemia progression and mice survival, the cells were isolated from spleens of untreated or DT-treated TCL1 leukemia-bearing mice (the same scheme of experiment as shown in **Figure 1A**), and adoptively transferred into TCL1-injected RAG2-KO mice (**Figure 2A**). Next, the expansion of TCL1 leukemic cells was monitored in murine blood twice a week. Interestingly, CD8^+^ T lymphocytes, isolated from Tregs-depleted mice effectively prevented leukemia progression, and in some mice even lead to complete elimination of TCL1 cells (**Figures 2B, C**). In contrast, the CD8^+^ T lymphocytes adoptively transferred from mice with intact Tregs population did not significantly affect the progression of the disease in RAG2-KO mice. Consequently, in TCL1-injected RAG2-KO mice, the adoptive transfer of CD8^+^ T cells isolated from DEREG mice after Tregs depletion, translated into prolonged survival and complete leukemia eradication in three out of nine mice. (**Figure 2D**).

The results obtained from the experiments described above revealed that the lack of Tregs in the leukemia microenvironment triggers the expansion of anti-leukemic CD8^+^ T cells. To address the differences in the investigated T cells, the CD8^+^ T cells from spleens of TCL1-injected DEREG mice treated with DT or untreated were sorted for DNA isolation and the T cell receptor beta chain (TCRβ) third complementarity-determining regions (CDR3) sequences analysis. An increase of CD8^+^ T cell clonality was observed in three out of five TCL1 leukemia-bearing mice with Tregs depletion, but overall, the observed differences were not statistically significant between the two examined groups (**Figure 2E**). Strikingly though, we observed distinct amino acid sequences of TCRβ CDR3 regions in the tested CD8^+^ T cells, suggesting different specificity of the T cells among untreated and DT-treated mice (**Figure 2F**). Indeed, only one sequence is shared in the top fifteen rearrangements between both analyzed CD8^+^ T cell populations (**Figure 2F**). Altogether, these data indicate that the elimination of Tregs from the TCL1 leukemia microenvironment resulted in the expansion of a distinct set of cytotoxic CD8^+^ T effector cells, capable of clearing leukemia in DEREG and RAG2-KO mice.

### CLL Leads to the Formation of a Specific Population of Tregs

**Figure 3:**
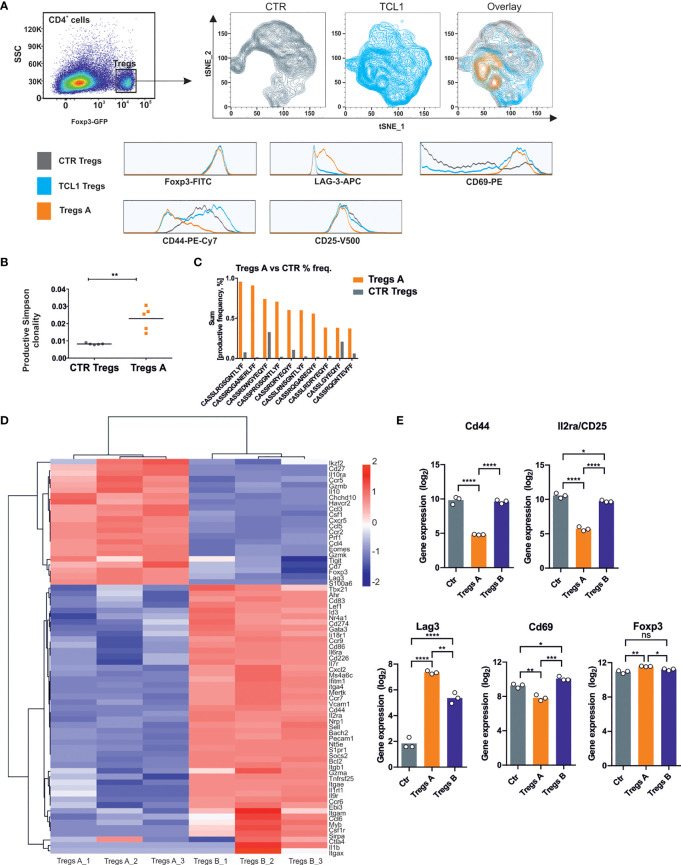
A specific Tregs population is formed during the progression of TCL1 leukemia. **(A)** tSNE analysis of Tregs phenotype isolated from control (grey) and TCL1 leukemia-bearing (blue) B6 Foxp3^EGFP^ mice 14 days after injections with leukemic cells. The overlay of counterplots presents the Tregs subpopulation, specific for TCL1 leukemia-bearing mice (Tregs A, orange). The Tregs GFP^+^-were plot on the graphs according to the expression of CD44, CD69, CD25, and LAG-3 that are presented on the histograms. The counter plots show representative analysis of Tregs from 2 control and 4 TCL1 leukemia-injected mice. **(B)** The productive Simpson clonality of Tregs sorted from control (all Tregs, CTR Tregs) and TCL1 leukemia-bearing (the specific TCL1-associated Tregs subpopulation, Tregs A) DEREG mice, n = 5. **(C)** The productive sum frequency of top 10 amino acid sequences of CDR3 TCRβ of Tregs A which were present in all of tested TCL1 leukemia-bearing DEREG mice, n = 5. **(D)** Clustering of selected DEGs between Tregs A and Tregs B (RNA sequencing with FDR < 0.05 and log_2_FC > 1) by correlation with complete linkage, n = 3. **(E)** Gene expression (log2, RNA sequencing) of genes from panel A in Tregs from CTR and TCL1 leukemia-bearing mice, n = 3. One-way ANOVA * p≤ 0.05, ** p< 0.01, *** p< 0.001.

We analyzed the phenotype and function of Tregs in TCL1-injected B6 FoxP3^EGFP^ transgenic mice that express *EGFP* and *FOXP3* under the control of endogenous promoter. Based on the results of phenotyping with a set of markers (FoxP3, LAG-3, CD69, CD44, CD25), we performed t-distributed stochastic neighbor embedding (tSNE) analysis, which allowed us to distinguish a specific Tregs subpopulation that exerts the phenotype characteristic only for Tregs isolated from TCL1 leukemia-bearing mice (Tregs A) (**Figure 3A**). This particular Tregs A subpopulation can be defined by high level of CD69, LAG-3 and low of CD44 and CD25 on their surface.

In order to investigate whether the observed changes in Tregs phenotype are mediated by the interactions with malignant B cells, we co-cultured the control Tregs-GFP^+^ (sorted from spleens of control B6 Foxp3^EGFP^ mice) with leukemic (TCL1) or normal (CD19) B cells. After three days, significantly higher level of LAG-3 was observed on Tregs-GFP^+^ co-cultured with TCL1 cells, but not with the control CD19^+^ cells (**Supplementary Figure 2A**). The elevated expression of LAG-3 was achieved only when Tregs-GFP+ and TCL1 leukemia cells were cultured in direct contact. On contrary, the level of CD44 in Tregs-GFP^+^ co-cultured with TCL1 leukemia cells (but not normal CD19^+^ cells) was reduced regardless the separation of the cells by transwells (**Supplementary Figure 2B**).

Next, the clonality of Tregs A subpopulation was examined, based on the TCRβ CDR3 region sequences. Importantly, the TCL1-associated Tregs A subpopulation sorted from spleens of TCL1-leukemia bearing DEREG mice exhibits increased clonality and elevated frequency of particular clones, compared to whole Treg-GFP^+^ subpopulation sorted from the control animals (CTR Tregs) (**Figures 3B, C**).

Finally, in order to characterize the TCL1-associated Tregs at the transcriptomic level, we performed RNA sequencing on two subpopulations of Tregs sorted from the spleens of TCL1-injected DEREG mice: Tregs A (specific to Eµ-TCL1 model, sorted as GFP^+^ CD44^-/lo^ and CD69^hi^) and Tregs B (the remaining GFP^+^ Tregs, which did not meet the criteria of subpopulation A). The transcriptome of both TCL1-associated subpopulations was compared with Tregs-GFP^+^ population sorted from spleens of control mice. Interestingly, the analysis of differentially expressed genes (DEGs), showed that Tregs A subpopulation was markedly different from both Tregs B as well as control Tregs populations (**Supplementary Figure 3, 4**). This data suggests that the specific Tregs A cells signature might be selectively induced within the conditions of leukemia progression. In Tregs A, we observed increased expression of genes responsible for immunosuppressive activity (*Gzmb, Prf1, Gzmk, Il10*), checkpoints (*Havcr 2, Lag-3, Tigit*), chemokines that may support leukemia progression and its microenvironment (*Ccl3, Csf1, Ccl5*), as well as genes that have been recently reported as unique for CLL-Tregs (*EOMES*) (**Figure 3D**). Importantly, the gene expression profile was in line with the phenotype observed in flow cytometry, apart from CD69, which seemed to be regulated post-transcriptionally (**Figure 3E**). Additionally, the elevated level of *Ikzf2* encoding Helios transcription factor suggests enhanced suppressive capacity of Tregs A subpopulation (27).

Next, we compared the gene expression profiles of Tregs A and B with a public dataset [GSE72494 (23)] describing the transcription profiles of naive, activated, and effector Tregs. We used a gene signature reported in that study (23), and built heat maps to compare Tregs subsets. The gene expression profiles indicated that our Ctrl Tregs population resembles naive Tregs and that Treg A and Tregs B exert similar expression patterns to effector Tregs and activated Tregs, respectively (**Supplementary Figure 4C**). Similar gene sets were identified as enriched in Tregs A (vs Treg B) and Effector Tregs (vs Activated Treg) (**Supplementary Tables 2** and **3**). Although Tregs A exhibited comparable transcription changes as compared to Effector Tregs (up-regulation of *Il10* and *Havcr2/Tim3* and down-regulation of *Sell* and *Ccr7*), we identified important differences suggesting a particular gene modulation in this specific Tregs population found in CLL (e.g. *Eomes, Prf1, Itgae, Cxcl10*) (**Supplementary Figure 4D**).

### The TCL1-Induced Tregs Are Functionally Active

**Figure 4:**
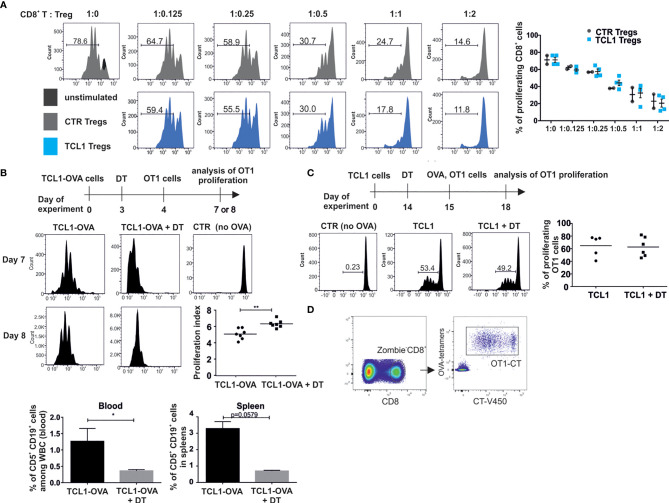
Tregs from TCL1 leukemia-bearing mice are capable of inhibiting T cells proliferation. **(A)** All Tregs-GFP^+^ sorted from spleens of control (CTR Tregs) and TCL1-injected (TCL1 Tregs) B6-Foxp3^EGFP^ mice, were added to the Cell Trace Violet (CT) stained CD8^+^ lymphocytes isolated from control mice and activated with αCD3 and αCD28 antibodies. The proliferation of – CT-stained CD8^+^ T cells was assessed by flow cytometry. Graphs show the results from two independent experiments, mean ± SD, n = 2-4 **(B)** OT1 CD8^+^ cell proliferation in mice injected with TCL1-OVA cells. After TCL1-OVA cells inoculation the mice were treated with DT and injected with CT-positive OT1 CD8^+^ lymphocytes (scheme of the experiment, upper panel). The proliferation of OT1 CD8^+^ cells from untreated or DT-treated mice was evaluated on the same day (7^th^ or 8^th^). The representative histograms of proliferation measured on days 7^th^ and 8^th^ are shown (left panel) and the proliferation index, from two independent experiments, is shown on the graph (middle right panel), n = 7 **p ≤ 0.01. Proliferation index was calculated by FlowJo software as the total number of divisions divided by the number of cells that went into division. The percentage of leukemic cells was assessed in blood and spleens of DEREG mice on day 8th of the experiment (lower panel), data is presented as mean ± SD, n = 3-4, Mann-Whitney U test * p ≤ 0.05. **(C)** OT1 CD8^+^ cell proliferation in mice injected with TCL1 cells and vaccinated with OVA protein. The representative histograms (left panel) and graph summarizing the results from two independent experiments (right panel), n = 5-6. **(D)** Gating strategy incorporated for analysis of CT-positive OT1 CD8^+^ T cells proliferation in functional *in vivo* tests.

Next, we determined the ability of splenic Tregs population, sorted from control and TCL1 leukemia-bearing B6 FoxP3^EGFP^ mice to inhibit CD8^+^ T cell proliferation in an antigen unspecific test, where T cells were activated *via* anti-CD3 and anti-CD28 antibodies. The obtained results indicated that whole Tregs population isolated from spleens of TCL1 leukemia-bearing mice is prone to inhibit CD8^+^ T cells proliferation similarly to control Tregs (**Figure 4A**). Similarly, in a test with OVA peptide presented by the bone marrow-derived dendritic cells, Tregs isolated from leukemic mice inhibited CD8^+^ OT1 cells proliferation as effectively as Tregs from control mice (**Supplementary Figure 5**). This data suggest that the effectiveness of antigen-independent suppression of Tregs from TCL leukemia-baring mice is similar to control Tregs.

In order to explore whether TCL1-associated Tregs suppress CD8^+^ T cells in an antigen specific manner, we generated OVA-expressing TCL1 by means of lentiviral transduction. DEREG mice were inoculated with TCL1-OVA cells, and 3 days later, Tregs were depleted with DT in one group. On the following day, mice were injected with Cell Trace (CT)-positive OT1 CD8^+^ T cells and the proliferation of these cells in the spleen was subsequently analyzed (**Figures 4B–D**). Interestingly, although the T cells were effectively activated in all tested TCL1 leukemia-bearing mice, in the group treated with DT, the proliferation was more efficient, suggesting that the Tregs population impeded OT1 CD8^+^ T cells proliferation to some extent. Moreover, a significant drop in the percentage of leukemic cells in blood and spleens of DT-treated mice was observed after injection of OT1 CD8^+^ T lymphocytes (**Figure 4B**). Conversely, when mice were inoculated with TCL1 cells (without OVA expression) and subsequently injected with OVA protein, no impact of Tregs depletion was observed on OT1 CD8^+^ T cells proliferation (**Figure 4C**). Altogether, these results suggest that Tregs inhibit proliferation of leukemia-specific CD8^+^ T cells in an antigen-dependent manner.

### Treatment With MALT1 Inhibitor Disturbs the Formation of Tregs A Subpopulation in TCL1 Leukemia-Bearing Mice and Enhances the Effect of Immunotherapy

**Figure 5:**
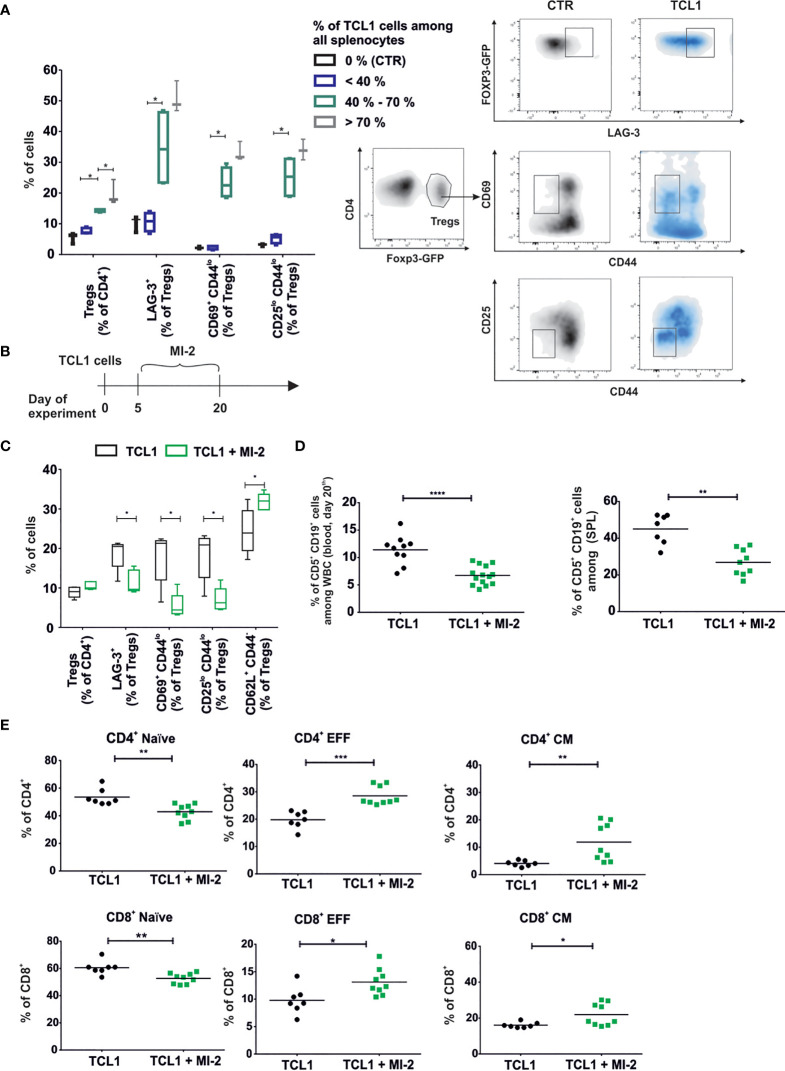
Development of a specific TCL1-related Tregs (Tregs A) population is correlated with the stage of the disease and can be blocked by MALT-1 inhibitor. **(A)** The box plot min-max graph (left panel) and density plots with gating strategy (right panel) present phenotype of Tregs in relation to the percentage of leukemic cells (CD5^+^CD19^+^) in the spleens. Mean ± SD, n=11, Mann-Whitney U test *p ≤ 0.05. **(B)** The graph presenting a scheme of the experiment. MI-2 was administered daily at dose 20 mg/kg *via* intraperitoneal injections for two weeks. **(C)** The phenotype of Tregs collected from spleens of TCL1 leukemia-bearing B6 Foxp3^EGFP^ mice, untreated (TCL1) or treated with MI-2(TCL1 + MI-2). Mean ± SD, Mann-Whitney U test n = 5, *p ≤ 0.05. **(D)** Percentage of leukemic cells (CD5^+^CD19^+^) assessed by flow cytometry in blood (left graph) and spleens (right graph) on day 21^st^ of the experiment. The graph presents data from three (blood, n = 10-14) or two (spleens, n = 7-8) independent experiments. Each dot represents an individual sample (mouse), means, Mann-Whitney U test **p ≤ 0.01, ***p ≤ 0.001, **** p < 0.0001. **(E)** The percent of naïve, effector (EFF), central memory (CM), subpopulations of CD4^+^ and CD8^+^ T cells. Cells were collected from spleens of untreated and MI-2-treated TCL1 leukemia-bearing B6 Foxp3^EGFP^ mice in two independent experiments, n = 7-12. Each dot represents an individual sample (mouse), means, Mann-Whitney U test *p ≤ 0.05, **p ≤ 0.01, ***p ≤ 0.001.

The analysis of Tregs phenotype at the various stages of leukemia revealed significant changes in the expression levels of Tregs surface proteins. The shift of Tregs into Tregs A phenotype escalated during leukemia progression and was accompanied by an increase in the percentage of splenic Tregs in leukemic mice (**Figure 5A**). Importantly, the Tregs A subpopulation was clearly formed at an advanced stage of the disease (when more than 40% of leukemic cells among all white blood cells were present in the spleens).

MI-2 has been described as a para-caspase MALT1 inhibitor that can selectively prevent the conversion of naïve Tregs into effector cells by decreasing the NFкB activity (21). MI-2 revealed its cytotoxic effect on primary CLL cells *in vitro* (28). Moreover, RNA sequencing analysis indicated elevated expression of NFкB-related genes in Tregs of TCL1-injected mice (**Supplementary Figure 4B**). In order to verify the influence of MI-2 on development of Tregs subpopulations, the inhibitor was administered intraperitoneally to the control and TCL1 leukemia-bearing B6 FoxP3^EGFP^ mice for two weeks starting from day 5 following TCL1 leukemic cells inoculation (**Figure 5B**). Administration of MI-2 impeded the change of Tregs into Tregs A phenotype and elevated the percentage of naïve Tregs (CD62L^+^ CD44^-^) (**Figure 5C**). MI-2 inhibited the progression of leukemia and increased significantly the percentage of central memory and effector CD4^+^ and CD8^+^ T lymphocytes (**Figures 5C–E**). Importantly, the effectiveness of MI-2 treatment was impaired in TCL1 leukemia-bearing RAG2-KO mice as compared to immunocompetent, wild type mice, suggesting a key role of T cells in the mechanism of action of this drug (**Supplementary Figures 6A, B**).

**Figure 6:**
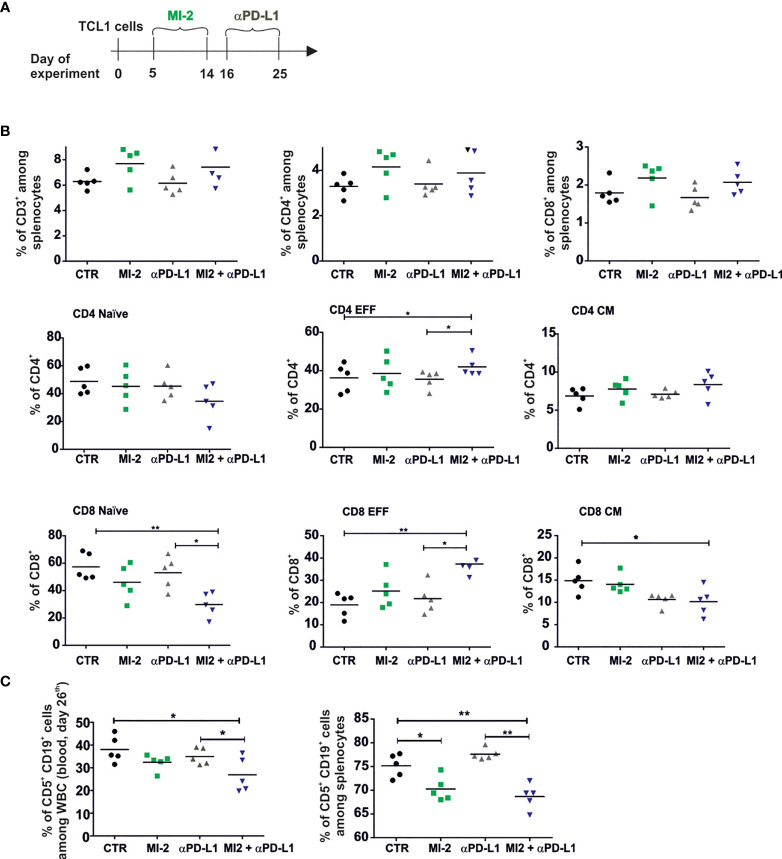
The pretreatment with MALT-1 inhibitor sensitizes leukemia to the therapy with anti-PD-L1 at the advanced stage of the disease. **(A)** The graphs presenting a scheme of the experiment. **(B)** The percentage of CD3^+^, CD4^+^, CD8^+^ and naïve, effector (EFF), and central memory (CM) subpopulations of CD4^+^ and CD8^+^. Cells were collected from spleens of untreated (CTR), MI-2 and/or aPD-L1-treated TCL1 leukemia-bearing mice, n = 4-5, each dot represents an individual sample (mouse), means, Mann-Whitney U test *p ≤ 0.05, **p ≤ 0.01. **(C)** The Percentage of leukemic cells (CD5^+^CD19^+^) assessed by flow cytometry in blood (left panel) and spleens (right panel) at day 26th of the experiment, n = 5, each dot represents an individual sample (mouse), means, Mann-Whitney U test *p ≤ 0.05, **p ≤ 0.01.

Since the PD1/PD-L1 axis was already shown to contribute to T cells dysregulations in both human and mouse models of CLL, we used MI-2 therapy as a pretreatment for checkpoint blockade with anti-PD-L1 antibody in immunocompetent TCL1-leukemia bearing mice (6, 29). Considering that long-term inhibition of Tregs functions can lead to autoimmune pathology (30), MI-2 inhibitor was used only before anti-PD-L1 therapy (**Figure 6A**). The anti-PD-L1 therapy did not affect the percentage of T cells already elevated by MI-2 (**Figure 6B**). However, the combined treatment decreased the percentage of naive cells and increased the percentage of effector cells of both CD4^+^ and CD8^+^ T lymphocytes (**Figure 6B**) Anti-PD-L1 antibodies administered 16 days post TCL1 inoculation decreased the percentage of leukemic cells in blood and spleen when applied after treatment with MI-2 (**Figure 6C**). These results indicate that the combination of Tregs inhibition with anti-PD-L1 antibody can bring beneficial treatment outcome in leukemia.

## Discussion

The anti-tumor strategy reducing the number of Tregs has been reported since 1999 (31). Nevertheless, targeting Tregs can yield differential responses in cancer models (32). In this study, we revealed that in the CLL mouse model, the depletion of Tregs population can lead to the expansion of CD8^+^ T cells with the ability to completely eradicate leukemia.

Published studies have consistently demonstrated elevated levels of Tregs in the peripheral blood collected from CLL patients compared to healthy subjects (33). The phenotype of analyzed Tregs was described as effector-like in both CLL patients and the Eμ-TCL1 mouse model of CLL (5, 16, 18). Our results indicate that the phenotype of Tregs changes during the course of leukemia to establish a subpopulation of CD4^+^, FoxP3^+^, LAG-3^+^, CD69^hi^, and surprisingly, CD44^lo^ and CD25^lo^ cells. A low expression of CD25 in Tregs has been already reported by another group (34), yet the CD44^lo^ phenotype is rather a characteristic feature of naïve lymphocytes. Our *ex vivo* experiments revealed that the level of cell-surface glycoprotein CD44 decreased in Tregs as a result of leukemia progression. At the transcriptomic level, however, the reduced amount of mRNA for CD44 was seen only in Tregs A, a specific TCL1-associated Tregs subpopulation distinguished for the first time in this study. Interestingly, the Tregs A subpopulation is positive for already reported markers of CLL-related Tregs, including IL-10, LAG-3, granzyme B, EOMES, as well as share a unique gene expression signature of chemokines that may support leukemia progression and formation of leukemic microenvironment (35, 36). Moreover, the overexpression of mRNA encoding HELIOS, TIGIT, TIM-3 and CD27 suggests that TCL1-related Tregs may possess immunosuppressive activity (27, 37–39). Importantly, our results prove that the observed change in the Tregs phenotype occurring during the progression of CLL results from the formation of a specific Tregs subpopulation (Tregs A).

Mpakou and colleagues show that Tregs isolated from CLL patients have an ability to inhibit CD8^+^ T cell proliferation (34). Likewise, according to our results, TCL1-derived Tregs are able to inhibit proliferation of T cells *ex vivo*. In *ex vivo* assays, T cells were activated in unspecific and specific manner, accordingly with the cognate antigen or by OVA peptide presented by dendritic cells thus the observed effect was not related to leukemia-specific antigens. The CLL-related Tregs functionality was finally confirmed in the *in vivo* experiment with TCL1-OVA cells, indicating that Tregs inhibit the proliferation of CD8^+^ cells upon recognition of tumor-expressed antigen.

The variable CDR3 regions of TCR interact with the peptide presented by MHC. The analysis of CDR3 sequence provides information about the diversity and clonality of investigated T cell populations and has become a valuable research tool in immunology (40). Thus, the higher oligoclonal composition of TCL1-derived Tregs compared to Tregs sorted from control mice, suggests that only selected clones of Tregs have undergone the expansion in TCL1 leukemia-bearing mice.

The expansion of exhausted T cells is a hallmark of human CLL and is also recapitulated in the Eµ-TCL1 mouse model (11). The CD8^+^ lymphocytes, which are present in spleens of TCL1 leukemia-bearing mice, have been described as antigen-experienced, oligoclonal cells that expand during the progression of the disease (12). In our experiments, upon depletion of Tregs, the CD8^+^ T cells became more oligoclonal and were effective in the elimination of leukemic cells. Surprisingly, anti-leukemic CD8^+^ T cells expressed different CDR3 sequences compared to the CDR3 sequences of lymphocytes from non-DT-treated, leukemia-bearing mice. It has been reported that in some tumors, based on the TCR sequences functional T cells formed a distinct group from dysfunctional, transitional tumor - infiltrating lymphocytes (41). Our results suggest that the depletion of Tregs in leukemia-bearing mice triggers the expansion of functional CD8^+^ T cell clones through the presentation of different epitopes than those used for splenic, exhausted CD8^+^ T cells. Elimination of Tregs primed the activation of T cells not only in spleens but also in the lymph nodes. The expansion of CD8^+^ T lymphocytes capable of killing leukemic cells occurred due to Tregs depletion, thus revealing their role in the maintenance of tumor antigen tolerance in CLL. The limitation of these studies is the fact that the antigens that led to the activation of anti-leukemic T lymphocytes were not identified yet. However, we suspect that these antigens could be associated with mutations typical for CLL. Importantly, we cannot rule out the possibility that these antigens are of other origins, for example derived due to genetic differences between mouse strains. Nevertheless, the depletion of Tregs seems to be a trigger for the expansion of effector T lymphocytes.

As it was also shown for other malignances, the inhibition of Tregs activation must occur early in the course of the disease to bring the beneficial outcome (42, 43). It has also been shown that the efficacy of adoptive T cell therapy is dependent on the tumor burden and is high in the early stages of tumor development or after chemotherapy (44, 45). To address this observation we conducted treatment with the MALT1 inhibitor, MI-2, when the leukemic cells were already detectable in blood but at a low level. MI-2 disrupted Tregs activation, prevented the formation of the specific TCL1-derived Tregs A subpopulation and inhibited the progression of leukemia in immunocompetent mice. Since the MI-2 was shown to exert a cytotoxic effect on leukemic cells (28), it is difficult to conclude from our experiments, whether it affects Tregs directly or only delays their activation due to the inhibition of leukemia progression. Though, the relatively small anti-leukemia efficacy of MI-2 obtained in RAG2-KO mice model may bring to the conclusion that T cells are important component in anti-leukemic MI-2 mechanism of action. Moreover, the decrease in the frequency of activated Tregs provided the therapeutic window to reduce the percentage of leukemic cells in mouse blood even two weeks after inoculation of leukemic cells.

Our results underline the role of Tregs in the progression of CLL and more importantly suggest that reactivation of the existing, exhausted T cell populations with anti-PD-L1 therapy, might be insufficient to block the disease progression. Notably, the presented results indicate that one approach to obtain an effective anti-leukemia immune response is to reorganize the CLL microenvironment, in order to create an opportunity for the expansion of a population of cytotoxic CD8^+^ T cells.

## Data Availability Statement

The datasets presented in this study can be found in online repositories. The names of the repository/repositories and accession number(s) can be found below: https://www.ncbi.nlm.nih.gov/, GSE179121.

## Ethics Statement

The animal study was reviewed and approved by The Local Ethics Committee for the Animal Experimentation in Warsaw, Warsaw University of Life Sciences, Warsaw, Poland.

## Author Contributions

Conceptualization, AM, AG, and MF. Investigation, AG, KF, MS, KS, PN, FS, GP, JP, and EM. Resources, JB, AS-P, SG, EL-M, and DE. Writing and visualization AM, AG, KF, JP, and EM. Critical revision, SG, FB, DE, PJ, MF, JP, and EM. Supervision, project administration AM. All authors contributed to the article and approved the submitted version.

## Funding

This work was supported by: the Polish National Science Centre grants 2018/29/B/NZ6/01962 (AM) and 2016/21/B/NZ7/02041 (MF) and the Ministry of Science and Higher Education within “Regional Initiative of Excellence” program in the years 2019-2022 - 013/RID/2018/19, the FNRS “Télévie” 7.6518.20 (GP), and the Fonds National de la Recherche Luxembourg TIME-CLL: C20/BM/14582635 (EM).

## Conflict of Interest

The authors declare that the research was conducted in the absence of any commercial or financial relationships that could be construed as a potential conflict of interest.

## Publisher’s Note

All claims expressed in this article are solely those of the authors and do not necessarily represent those of their affiliated organizations, or those of the publisher, the editors and the reviewers. Any product that may be evaluated in this article, or claim that may be made by its manufacturer, is not guaranteed or endorsed by the publisher.
