# Supplementary material for: A Specific CD44lo CD25lo Subpopulation of Regulatory T Cells Inhibits Anti-Leukemic Immune Response and Promotes the Progression in a Mouse Model of Chronic Lymphocytic Leukemia
Source: Front Immunol. 2022 Feb 28;13:781364. doi: 10.3389/fimmu.2022.781364 (PMC8918500; doi:10.3389/fimmu.2022.781364)
Supplement: Supplementary file 1 [file DataSheet_1.pdf]

frontiers

Supplementary Material

1 Supplementary Figures and Tables

1.1 Supplementary Figures

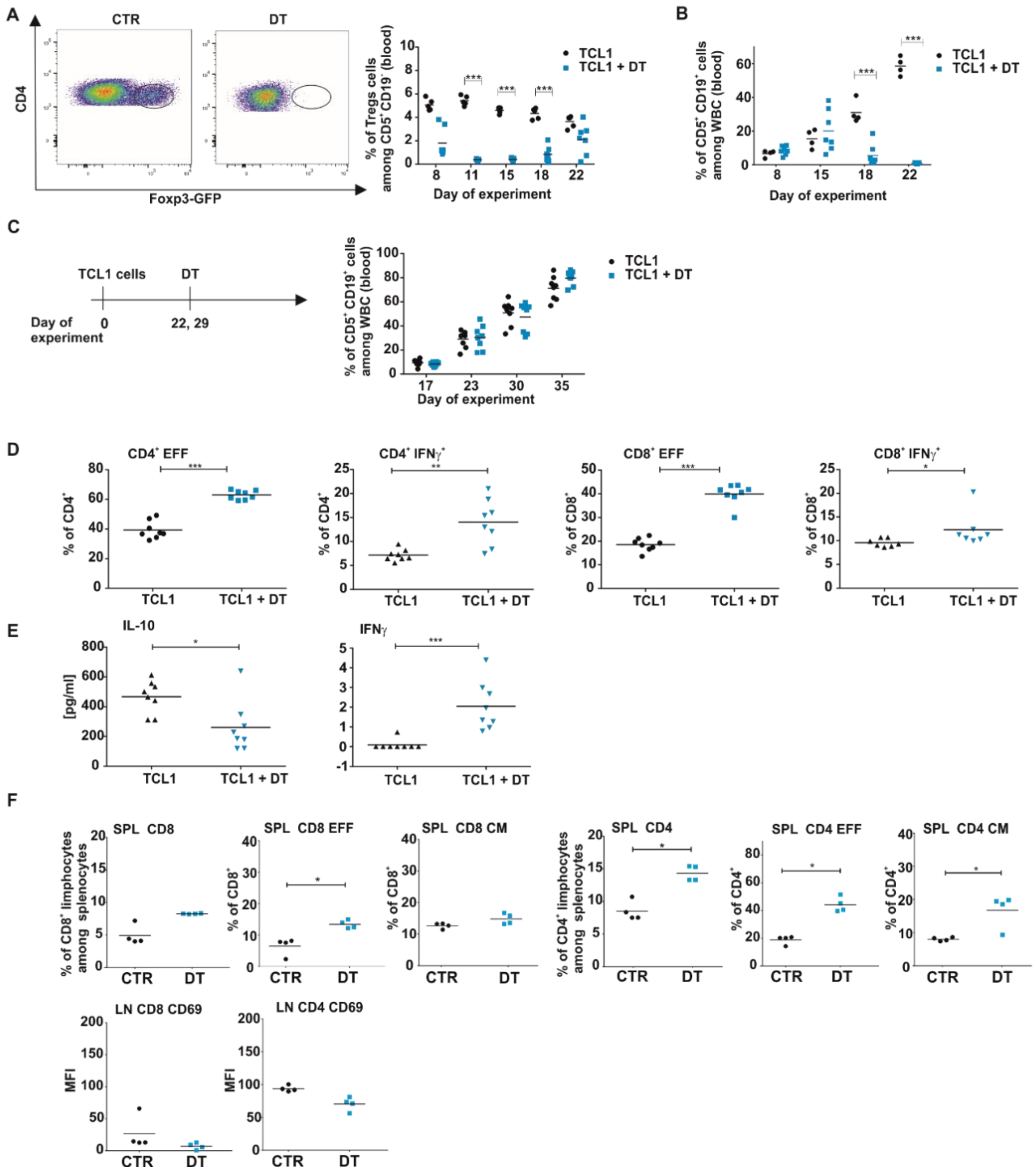

Supplementary Figure 1. (**A**) The representative dot plots showing the efficacy of Tregs depletion in DEREG mice spleens 24 hours upon DT injection (left) and graph presenting the percentage of Tregs (among CD5+ cells) assessed by flow cytometry at indicated time points in peripheral blood collected from untreated and DT-treated TCL1 leukemia-bearing DEREG mice (right panel). Each dot represents an individual sample (mouse), n=4-7, mean $*p \leq 0.05$, $***p \leq 0.001$.

(**B**) Percentage of leukemic cells ($CD5^{+}CD19^{+}$) assessed with flow cytometry in blood collected from untreated and DT-treated TCL1 leukemia-bearing DEREG mice a different batch of TCL1 leukemic

cells were used (isolated from Eμ-TCL1-1013 transgenic mouse) than those presented on Figure 1, Each dot represents an individual sample (mouse) n=4-7, ***$p \leq 0.001$.

(**C**) Scheme of the experiment showing different time points for Tregs depletion as compared to data presented on Figure 1 (left panel) and graph presenting the percentage of leukemic cells ($CD5^{+}CD19^{+}$) assessed by flow cytometry at indicated time points in blood collected from untreated and DT-treated TCL1 leukemia-bearing DEREG mice, each dot represents an individual sample (mouse), n=8.

(**D**) The percentage of effector (EFF) and IFN-γ^{+} subpopulations of $CD4^{+}$ and $CD8^{+}$ T cells. Cells were collected from spleens of untreated and DT-treated TCL1 leukemia-bearing DEREG mice and stained for CD44 and CD62L, each dot represents an individual sample (mouse), n=8, *$p \leq 0.05$, **$p \leq 0.01$, ***$p \leq 0.001$.

(**E**) The analysis of IL-10 and IFN- γ concentrations in mouse serum, collected from untreated and DT-treated TCL1-injected DEREG mice, each dot represents an individual sample (mouse), n=8, *$p \leq 0.05$, ***$p \leq 0.001$.

(**F**) The percentage of $CD8^{+}$ and $CD4^{+}$, effector (EFF) and central memory (CM) subpopulations in spleens (upper panel) and CD69 level in $CD8^{+}$ and $CD4^{+}$ of lymph nodes (lower panel) of DEREG control and DT-treated mice (both without leukemia), each dot represents an individual sample (mouse) n=4, *$p \leq 0.05$.

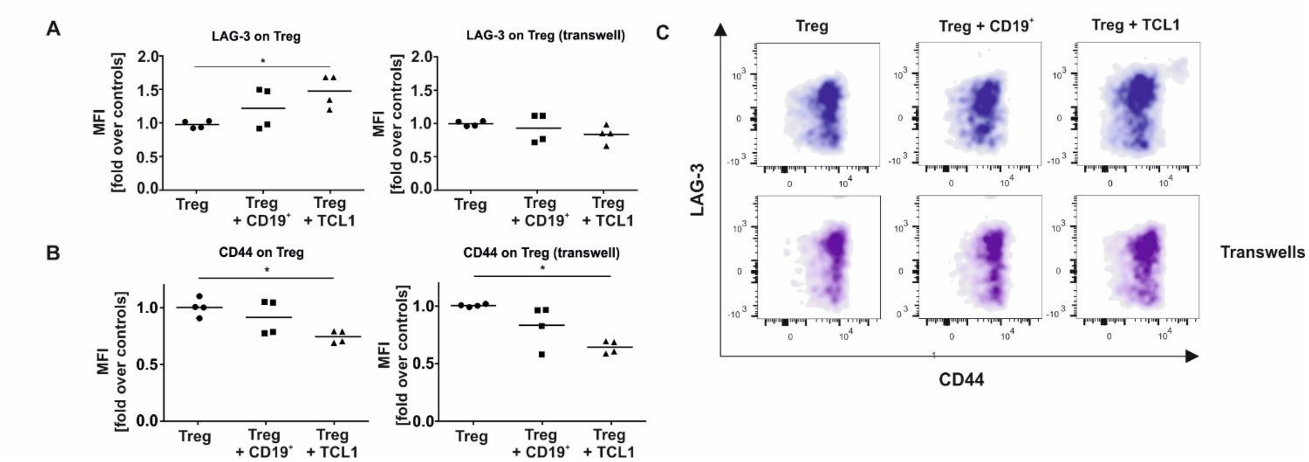

Supplementary Figure 2. The expression of LAG-3 (**A**) or CD44 (**B**) and representative density plots (**C**) of Tregs sorted and co-cultured *ex vivo* with $CD19^+$ isolated from control ($CD19^+$) and TCL1 leukemia-bearing (TCL1) mice. Cells were mixed (left graphs) or separated by transwells (right graphs) and cultured for 72 hours. The graphs show results from two independent experiments, mean ±SD, $p \leq 0.05$.

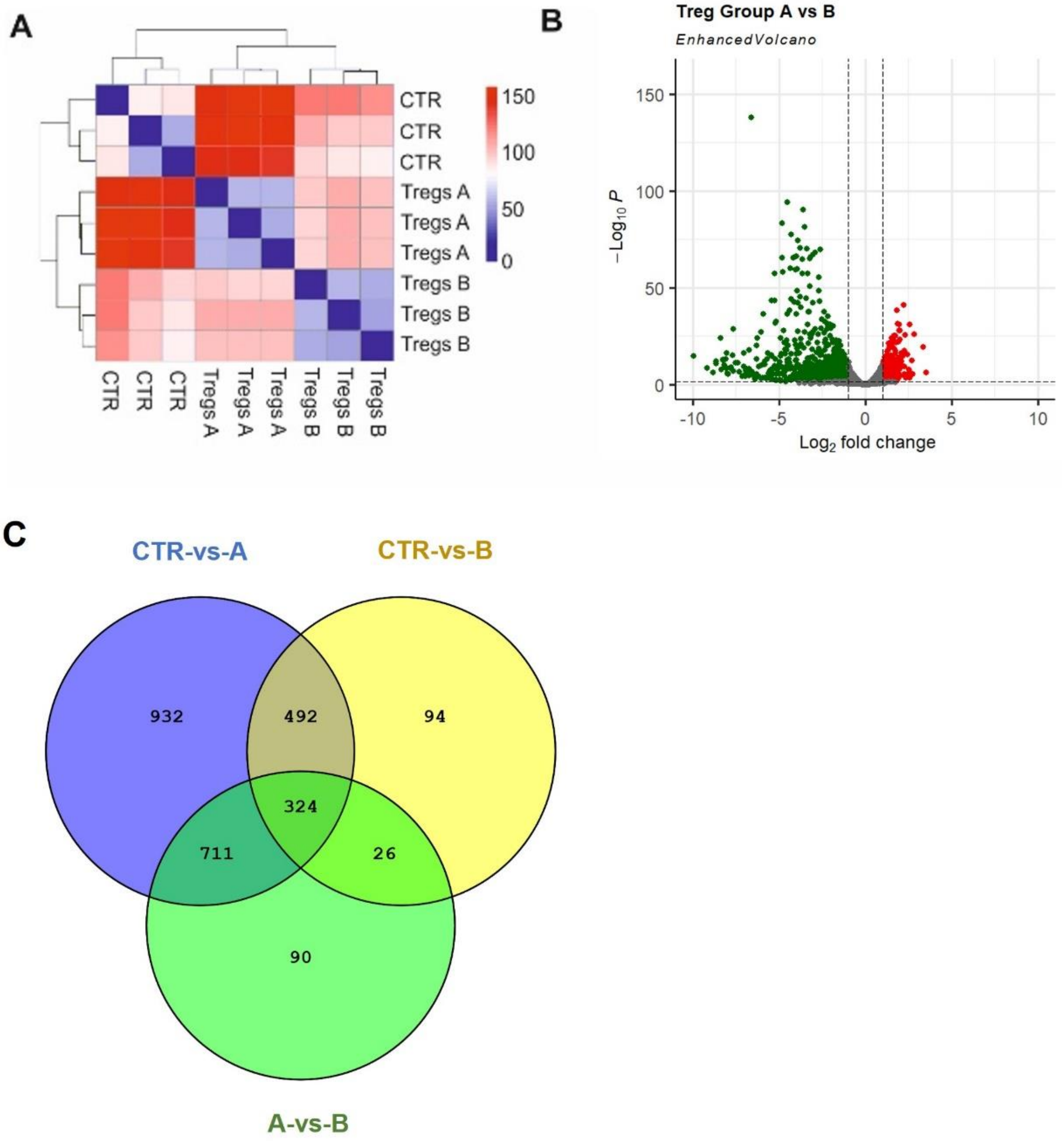

Supplementary Figure 3. (**A**) The Euclidean distance was used to assess the overall similarity between samples. Log2-transformed gene expressions were used to compute distances in a matrix that were visualized in a heatmap. CTR and Tregs A appear as the most different conditions.
(**B**) Volcano plot showing gene expression comparison between Tregs A and Tregs B. Most of DEGs are down-regulated in Tregs A vs Tregs B from TCL1-bearing mice.
(**C**) Venn diagram indicating the number of DEGs common between the comparisons. Most DEGs belonged to the CTR-Tregs A comparison, confirming these conditions as the most different.

A

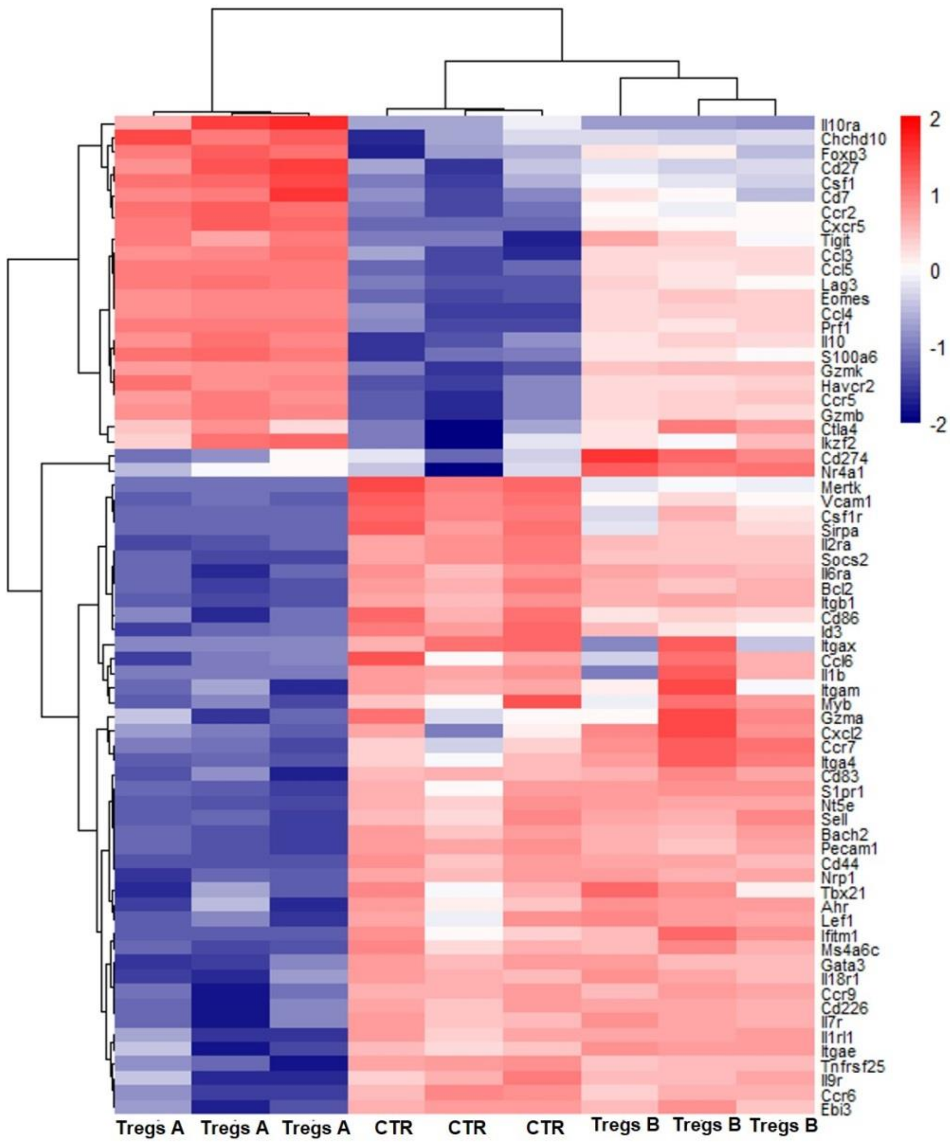
Il10ra
Chchd10
Foxp3
Cd27
Csf1
Cd7
Ccr2
Cxcr5
Tigit
Ccl3
Ccl5
Lag3
Eomes
Ccl4
Prf1
Il10
S100a6
Gzmk
Havcr2
Ccr5
Gzmb
Ctla4
Ikzf2
Cd274
Nr4a1
Mertk
Vcam1
Csf1r
Sirpa
Il2ra
Socs2
Il6ra
Bcl2
Itgb1
Cd86
Id3
Itgax
Ccl6
Il1b
Itgam
Myb
Gzma
Cxcl2
Ccr7
Itga4
Cd83
S1pr1
Nt5e
Sell
Bach2
Pecam1
Cd44
Nrp1
Tbx21
Ahr
Lef1
Ifitm1
Ms4a6c
Gata3
Il18r1
Ccr9
Cd226
Il7r
Il1rl1
Itgae
Tnfrsf25
Il9r
Ccr6
Ebi3
2
1
0
-1
-2
Tregs A
Tregs A
Tregs A
CTR
CTR
CTR
Tregs B
Tregs B
Tregs B

B

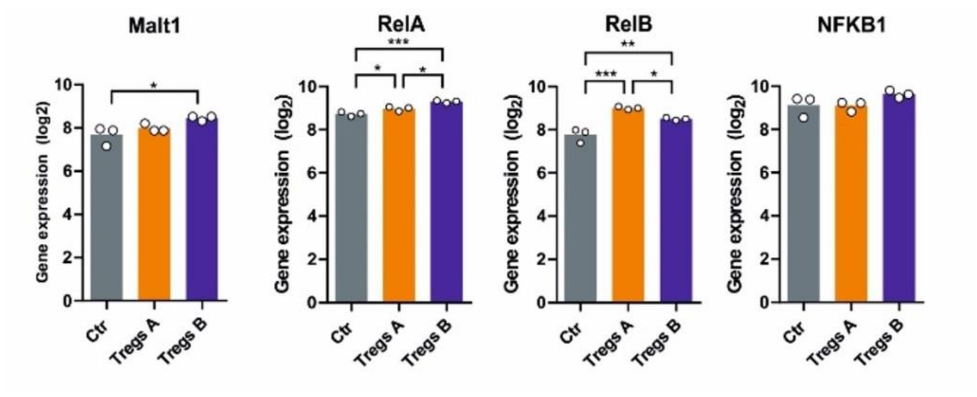
Malt1
RelA
RelB
NFKB1
Gene expression (log2)
Gene expression (log_2)
*
***
**
0
2
4
6
8
10
Ctr
Tregs A
Tregs B

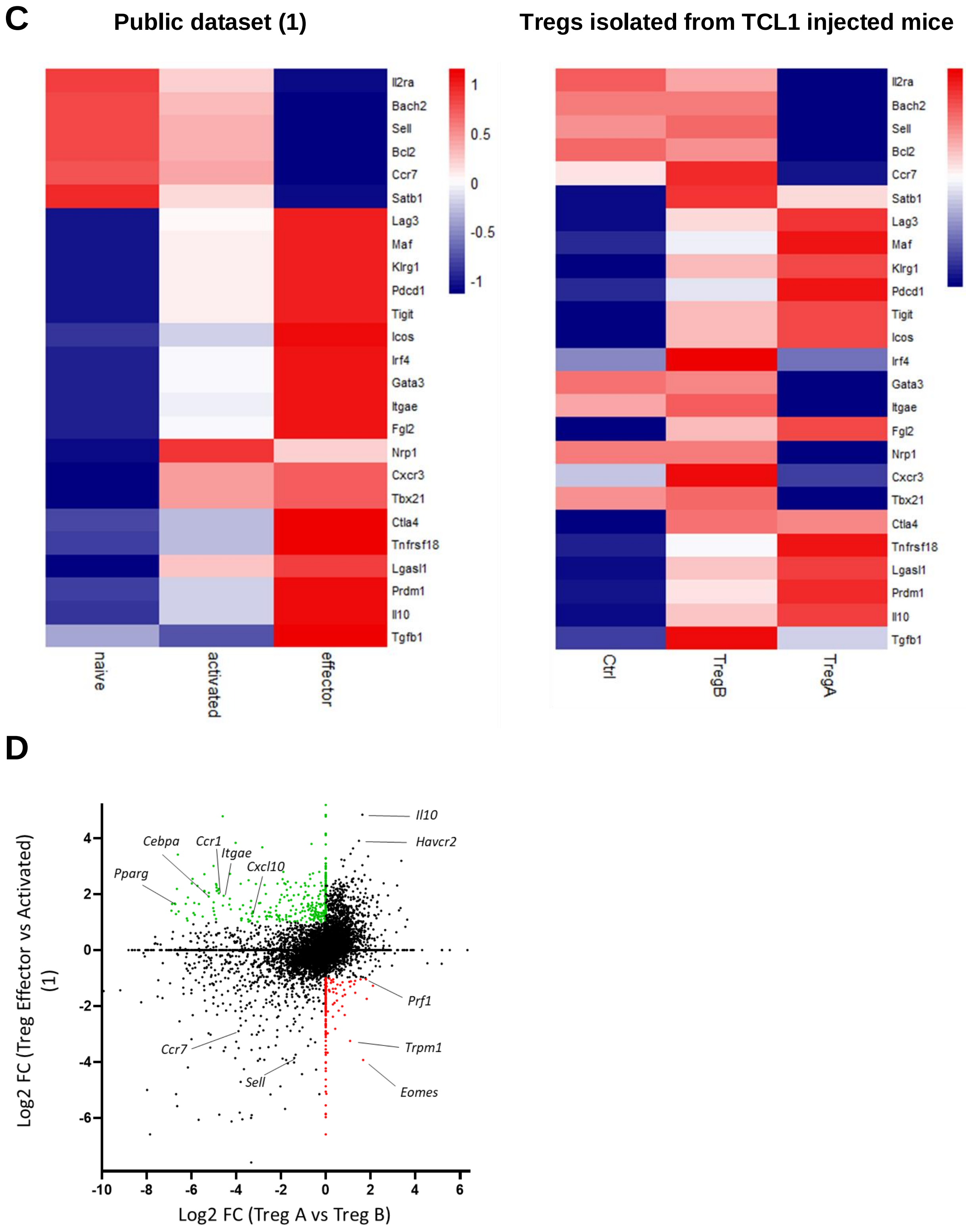

Supplementary Figure 4. (**A**) Clustering of selected DEGs in Tregs from control and TCL1-bearing mice (RNA sequencing with FDR < 0.05 and log2FC > 1) by correlation with complete linkage, n=3 for each Tregs subpopulation.

(**B**) The expression (log2, RNA sequencing) of genes regulating the NF-κB pathway in Tregs from control and TCL1-bearing mice, n=3. One-way ANOVA * p≤ 0.05, ** p< 0.01, *** p<0.001.

(**C**) Heatmaps showing the expression of genes selected from a publicly available dataset ((1), GSE72494) in naïve, activated and effector Tregs (left panel) and from our RNA-seq data among control, Tregs A, and Tregs B subpopulations isolated from TCL1 injected DEREG mice (described in details in Figure 3).
(**D**) Plot showing gene expression comparison between effector vs activated Tregs (GSE72494), and Tregs A vs Tregs B. Green dots represent genes upregulated in effector vs activated Tregs (log FC>1) and downregulated (or not different) in Tregs A vs Tregs B (total of 442 genes). Red dots represent genes downregulated in effector vs activated Tregs (logFC<-1) and downregulated (or not different) in Tregs A vs Tregs B (total of 172 genes). Black dots represent genes that moves similarly between the 2 comparisons..

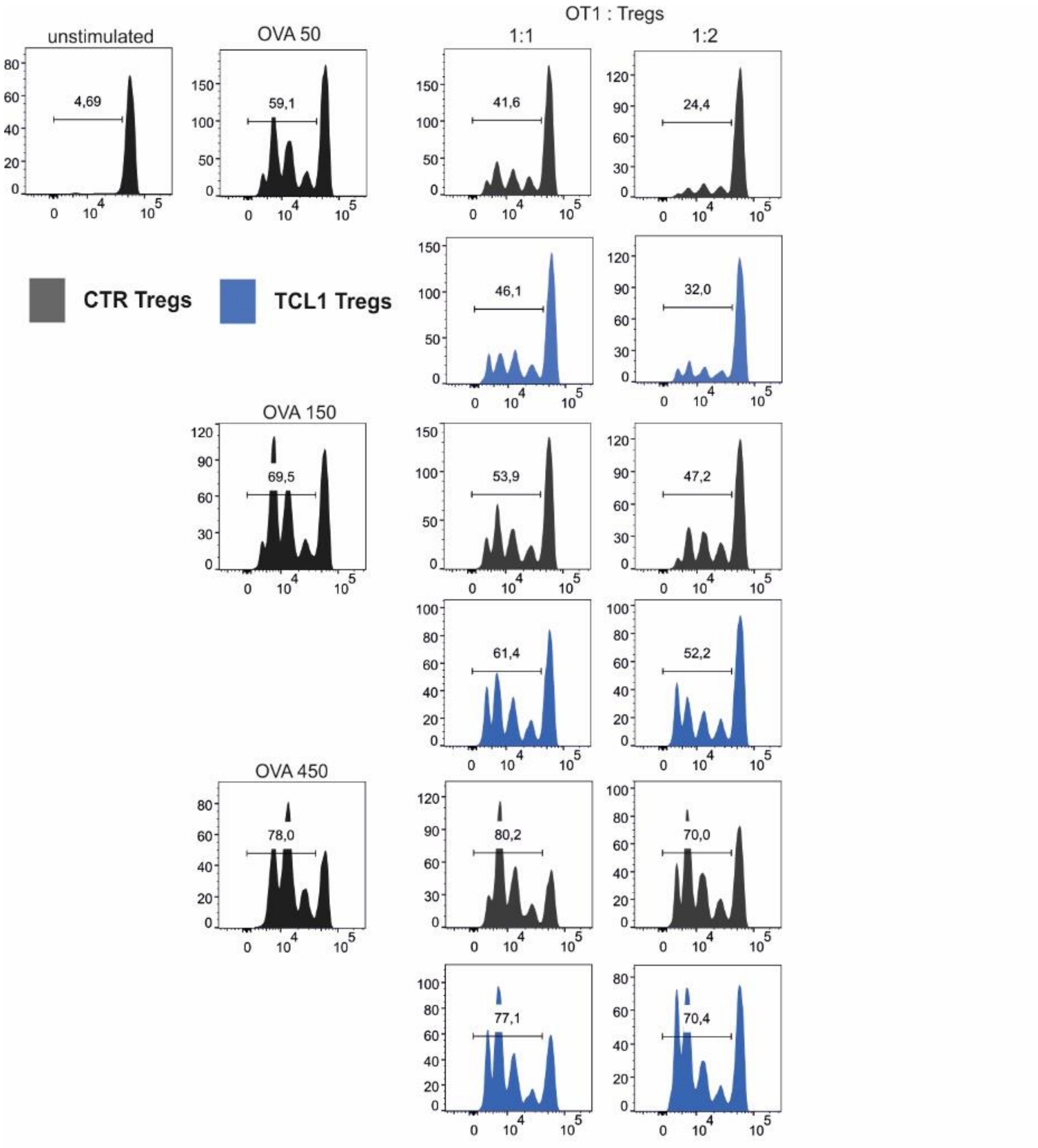

Supplementary Figure 5. OT1 T cell proliferation primed by bone marrow derived-dendritic cells after incubation with OVA peptide (50, 150, 450 μM). Dendritic cells were co-cultured with Tregs, sorted from control and TCL1-injected B6 Foxp^{EGFP} mice. Subsequently, the OT1 CD8^{+} cells stained with Cell Trace Violet were added and their proliferation was assessed with flow cytometry after 72 hours.

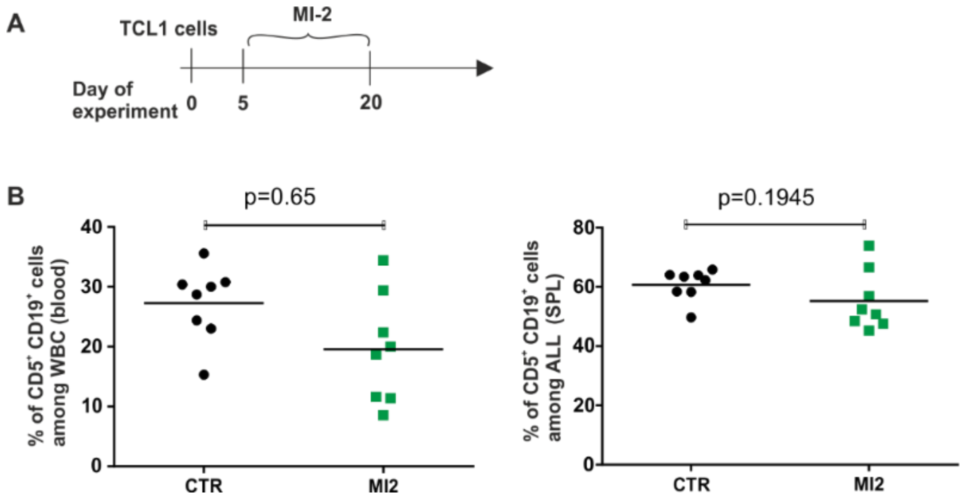

Supplementary Figure 6. (**A**) Scheme of experiment. RAG2-KO were injected with TCL1 cells and treated with MI-2 for two weeks at dose 20 mg/kg.
(**B**) Percentage of leukemic cells ($CD5^+CD19^+$) assessed with flow cytometry in blood collected from untreated and MI-2-treated TCL1 leukemia-bearing RAG2-KO mice. The experiment was repeated twice, each dot represents an individual sample (mouse) n=7-8.

1.2 Supplementary Tables

Supplementary Table 1. Antibodies used in this study

ANTIBODY	SOURCE	IDENTIFIER
BD Pharmingen™ PE Rat Anti-Mouse CD5, clone: 53-7.3	BD Biosciences	Cat# 553023; RRID:AB_394561
BD Pharmingen™ APC Rat Anti-Mouse CD19; clone: 1D3	BD Biosciences	Cat# 550992; RRID:AB_398483
CD3 Monoclonal Antibody (17A2), eFluor 450	eBioscience/Thermo Fisher Scientific	Cat# 48-0032-82; RRID:AB_1272193
BD Pharmingen™ APC-Cy™7 Rat Anti-Mouse CD4, clone: GK1.5	BD Biosciences	Cat#: 552051; RRID:AB_394331
BD Pharmingen™ PE-Cy™7 Rat Anti-Mouse CD4, clone: GK1.5	BD Biosciences	Cat#: 563933; RRID:AB_2738492
CD8a Monoclonal Antibody (53-6.7), PerCP-Cyanine5.5	eBioscience/Thermo Fisher Scientific	Cat#: 45-0081-82
BD Horizon™ BV510 Rat Anti-Mouse CD25, clone: C61	BD Biosciences	Cat#: 563037; RRID:AB_2737969
CD44 Monoclonal Antibody (IM7), PE-Cyanine7	eBioscience/Thermo Fisher Scientific	Cat#: 25-0441-81; RRID:AB_469622
CD62L (L-Selectin) Monoclonal Antibody (MEL-14), APC	eBioscience/Thermo Fisher Scientific	Cat#: 17-0621-81; RRID:AB_469409
CD69 Monoclonal Antibody (H1.2F3), PE	eBioscience/Thermo Fisher Scientific	Cat#: 12-0691-81; RRID:AB_465731
PerCP-Cy™5.5 Rat Anti-Mouse Ly-6C, clone: AL-21	BD Biosciences	Cat#: 560525; RRID:AB_1727558
BD Horizon™ V450 Rat Anti-Mouse Ly-6C, clone: AL-21	BD Biosciences	Cat#: 560594; RRID:AB_1727559
BD Pharmingen™ PE Rat Anti-Mouse IFN-γ, clone: XMG1.2	BD Biosciences	Cat#: 554412; RRID:AB_395376
BD Pharmingen™ APC Rat Anti-Mouse CD223, clone: C9B7W	BD Biosciences	Cat#: 562346
BD Pharmingen™ PerCP-Cy™5.5 Rat Anti-Mouse CD223, clone: C9B7W	BD Biosciences	Cat#: 564673, RRID:AB_2734764
Brilliant Violet 421™ anti-mouse CD274 (B7-H1, PD-L1) Antibody, clone: 10F.9G2	BioLegend	Cat#: 124315; RRID:AB_10897097
BD Pharmingen™ APC Rat Anti-Mouse IL-10, clone: JES5-16E3	BD Biosciences	Cat#: 554468; RRID:AB_398558
CD3e Monoclonal Antibody (145-2C11), Functional Grade	eBioscience/Thermo Fisher Scientific	Cat#: 16-0031-85; RRID:AB_468848
CD28 Monoclonal Antibody (37.51), Functional Grade	eBioscience/Thermo Fisher Scientific	Cat#: 16-0281-85; RRID:AB_468922
InVivoPlus anti-mouse PD-L1 (B7-H1), clone: 10F.9G2	BioXcell	Cat#: BP0101; RRID:AB_10949073
InVivoPlus rat IgG2b isotype control, anti-keyhole limpet hemocyanin, clone: LTF-2	BioXcell	Cat#: BP0090; RRID:AB_1107780

Supplementary Table 2. Gene Set Enrichment Analysis (GSEA) of Tregs A and Tregs B gene expression.

Tregs A	GeneSet	NES	NOM p-val
	GAVIN_FOXP3_TARGETS_CLUSTER_P6	1,91	0,000
	MARSON_FOXP3_CORE_DIRECT_TARGETS	1,8	0,007
	HALLMARK_E2F_TARGETS	1,73	0,000
	HALLMARK_G2M_CHECKPOINT	1,58	0,000
	WP_OXIDATIVE_PHOSPHORYLATION	1,39	0,028
	HALLMARK_MTORC1_SIGNALING	1,36	0,000
	MARSON_FOXP3_TARGETS_UP	1,35	0,031
	KEGG_OXIDATIVE_PHOSPHORYLATION	1,32	0,000
	HALLMARK_CHOLESTEROL_HOMEOSTASIS	1,29	0,049

Tregs B	GeneSet	NES	NOM p-val
	GAVIN_FOXP3_TARGETS_CLUSTER_P7	-1,8	0,000
	ZHENG_FOXP3_TARGETS_IN_T_LYMPHOCYTE_DN	-1,76	0,000
	GAVIN_FOXP3_TARGETS_CLUSTER_T4	-1,68	0,000
	ZHENG_BOUND_BY_FOXP3	-1,67	0,000
	ZHENG_FOXP3_TARGETS_IN_THYMUS_UP	-1,66	0,000
	HALLMARK_TNFA_SIGNALING_VIA_NFKB	-1,65	0,000
	HALLMARK_IL6_JAK_STAT3_SIGNALING	-1,63	0,000
	HALLMARK_TGF_BETA_SIGNALING	-1,61	0,003
	HALLMARK_INFLAMMATORY_RESPONSE	-1,58	0,000
	HALLMARK_IL2_STAT5_SIGNALING	-1,56	0,000
	HALLMARK_INTERFERON_GAMMA_RESPONSE	-1,38	0,002
	HALLMARK_HYPOXIA	-1,33	0,011

Supplementary Table 3. Gene Set Enrichment Analysis (GSEA) of Effector Tregs and Activated Tregs gene expression (1).

Effector	GeneSet	NES	NOM p-val
	FISCHER_G2_M_CELL_CYCLE	2,43	0
	KONG_E2F3_TARGETS	2,43	0
	GAVIN_FOXP3_TARGETS_CLUSTER_P6	2,36	0
	HALLMARK_G2M_CHECKPOINT	2,25	0
	HALLMARK_E2F_TARGETS	2,24	0
	HALLMARK_MITOTIC_SPINDLE	1,73	0
	GAVIN_FOXP3_TARGETS_CLUSTER_P3	1,54	0,003
	HALLMARK_MTORC1_SIGNALING	1,47	0,003
	HALLMARK_GLYCOLYSIS	1,36	0,019
	HALLMARK_P53_PATHWAY	1,34	0,025

	HALLMARK_TNFA_SIGNALING_VIA_NFKB	1,33	0,025
	HALLMARK_IL2_STAT5_SIGNALING	1,27	0,048

**Active**	**GeneSet**	**NES**	**NOM p-val**
	ZHENG_FOXP3_TARGETS_IN_T_LYMPHOCYTE_DN	-2,13	0
	GAVIN_FOXP3_TARGETS_CLUSTER_P7	-1,93	0
	ZHENG_BOUND_BY_FOXP3	-1,48	0
	GAVIN_FOXP3_TARGETS_CLUSTER_P2	-1,47	0,007
	GAVIN_FOXP3_TARGETS_CLUSTER_P4	-1,42	0,011
	GAVIN_FOXP3_TARGETS_CLUSTER_T7	-1,39	0,028

2 Supplementary Methods

2.1 Cell culture

Murine bone marrow stromal M2-10B4 and human embryonic kidney (HEK 293T) cell lines were purchased from American Type Culture Collection (ATCC) (Manassas, VA, USA) The genetic characteristics of HEK293T cells was determined by PCR-single-locus-technology in 2020. The cells were cultured in Roswell Park Memorial Institute (RPMI-1640) medium (Gibco/Thermo Fisher Scientific, Waltham, MA, USA) or Dulbecco's Minimal Eagle's Medium (DMEM) (Sigma Aldrich, St Louis, MA, USA), respectively. Mouse hybridoma cell line Sp2.0 transfected with gene for soluble Fms Related Receptor Tyrosine Kinase 3 Ligand (FLT3L secreting cells) (2), were cultured in Iscove's Modified Dulbecco's Medium (IMDM) (Gibco/Thermo Fisher Scientific, Waltham, MA, USA). All media were supplemented with 10% (v/v) heat-inactivated fetal bovine serum (FBS) (HyClone Laboratories, Logan, Utah, USA) gentamycin (50 µg/ml) (Sigma-Aldrich, St Louis, MA, USA) and cells were cultured at 37°C, 5% CO_2, in a humidified atmosphere. For *ex vivo* experiments, the medium was supplemented with 2 mM L-glutamine (Sigma-Aldrich, St Louis, MA, USA) and 50 µM B-mercaptoethanol (Gibco/Thermo Fisher Scientific, Waltham, MA, USA). The cell lines were tested for Mycoplasma spp. contamination once a week.

2.2. Co-cultures of Tregs and CD19/TCL1 cells

M2-10B4 murine stroma cells were seeded onto 24-well plates at the density 5.0×10^3 cell per well. Next day, B cells isolated from spleens of control ($CD19^+$) and leukemia bearing mice (TCL1) were seeded into wells (1.0×10^6 cells per well). Then Tregs sorted from spleens of control FoxP3-GFP mice were added to the wells (1.5×10^4 cells /well) directly or seeded on cell culture inserts (FALCON/Corning, Corning, NY, USA). For phenotyping of Tregs, upon three days of co-culture the cells were stained with proper antibodies and analysed by flow cytometry.

2.3. Mouse serum preparation and assessment of cytokine concentration in mouse serum

Blood samples were collected from cheek vein into anticoagulant-free tubes and left for 1h at RT to form a cloth. Then the tubes were centrifuged at 1000 x g for 10 min. Collected serum was aliquoted and stored at -80°C. BD Cytometric Bead Array (CBA) Mouse Th1/Th2/Th17 CBA Kit (BD Biosciences) was applied according to the manufacturer's protocol and analysed by flow cytometry .

2.4. Dendritic cells differentiation

Bone marrow immune cells were isolated from femurs and tibia of 5-6 weeks old, wild type C57BL6/J mice and dendritic cells (DCs) were differentiated as described before (3). Briefly, the isolated bone marrow cells were seeded onto non-tissue culture treated 6-well plates at the density 1.0×10^6/ml and cultured in IMDM medium (Gibco/Thermo Fisher Scientific, Waltham, MA, USA) supplemented with 10% (v/v) heat-inactivated FBS (HyClone Laboratories,) gentamycin (50 µg/ml) (Sigma-Aldrich,), 50 µM β-mercaptoethanol (Gibco/Thermo Fisher Scientific,) and 15% of FLT3L-containing supernatant (complete IMDM medium) at 37°C, 5% CO_2, in a humidified atmosphere for 7 days. At day 3rd and 6th the half volume of the medium was removed and replaced by the complete fresh medium. DCs were used for priming experiments between 7th and 9th day.

2.5. Priming assay

Bone marrow-derived DCs were seeded onto tissue treated 96-well plates (2.0×10^4 cells/well) in complete IMDM medium supplemented with Poly(I:C)(HMW) (1 µg/ml) (InvivoGen, San Diego, CA, USA), pulsed with OVA protein (Albumin from chicken egg white, Sigma-Aldrich) (50, 150 or 450 µg) for 3 hours and washed twice with RPMI medium supplemented with 10% (v/v) heat-inactivated FBS (HyClone Laboratories,), gentamycin (50 µg/ml) (Sigma-Aldrich), 50 µM B-mercaptoethanol (Gibco/Thermo Fisher Scientific) and 2 mM L-glutamine (Sigma-Aldrich). Next, Tregs sorted as described in main text, from spleens of control or TCL1 leukemia-bearing B6 $Foxp3^{EGFP}$ mice were added to the proper wells together with 4×10^4 cells/well) $CD8^+$ T cells isolated by magnetic separation (EasySep™ Mouse $CD8^+$ Cell Isolation Kit; STEMCELL Technologies) from spleens of OT1 mice and stained with CT as described above. The $CD8^+$: Tregs-GFP ratio was 1:1 or 1:2. The proliferation of $CD8^+$ cells was evaluated upon 72 hours using flow cytometry.

Supplementary References

1. Dias S, D'Amico A, Cretney E, Liao Y, Tellier J, Bruggeman C, et al. Effector Regulatory T Cell Differentiation and Immune Homeostasis Depend on the Transcription Factor Myb. Immunity. 2017;46(1):78-91.

2. Dehlin M, Bokarewa M, Rottapel R, Foster SJ, Magnusson M, Dahlberg LE, et al. Intra-articular fms-like tyrosine kinase 3 ligand expression is a driving force in induction and progression of arthritis. PLoS One. 2008;3(11):e3633.

3. Caronni N, Simoncello F, Stafetta F, Guarnaccia C, Ruiz-Moreno JS, Opitz B, et al. Downregulation of Membrane Trafficking Proteins and Lactate Conditioning Determine Loss of Dendritic Cell Function in Lung Cancer. Cancer Res. 2018;78(7):1685-99.
